# Fear of Falling Contributing to Cautious Gait Pattern in Women Exposed to a Fictional Disturbing Factor: A Non-randomized Clinical Trial

**DOI:** 10.3389/fneur.2019.00283

**Published:** 2019-03-26

**Authors:** Guilherme Augusto Santos Bueno, Flávia Martins Gervásio, Darlan Martins Ribeiro, Anabela Correia Martins, Thiago Vilela Lemos, Ruth Losada de Menezes

**Affiliations:** ^1^Postgraduate Program in Health Sciences and Technologies, University of Brasília, Brasília, Brazil; ^2^Movement Laboratory Dr. Cláudio A. Borges, College of Sport, State University of Goiás, Goiânia, Brazil; ^3^Dr. Henrique Santillo Rehabilitation and Readaptation Center, Goiânia, Brazil; ^4^Department of Physiotherapy, ESTeSC - Coimbra Health School, Polytechnic Institute of Coimbra, Coimbra, Portugal

**Keywords:** aging, accidental falls, perception, motor skills, biomechanical phenomena

## Abstract

**Objective:** This study aimed to investigate the gait pattern of elderly women with and without fall-history, with high and low fear of falling, when exposed to a disturbing factor.

**Materials and Methods:** Forty-nine elderly women without cognitive impairment agreed to participate. Participants were divided into four groups, considering the history of falls and fear of falling. Three-dimensional gait analysis was performed to assess gait kinematics before and after exposure to the fictional disturbing factor (psychological and non-motor agent).

**Results:** After being exposed to the perturbation, all showed shorter step length, stride length and slower walking speed. Those without fall-history and with high fear of falling showed greater changes and lower Gait Profile Score.

**Conclusion:** The gait changes shown in the presence of a fear-of-falling causing agent led to a cautious gait pattern in an attempt to increase protection. However, those changes increased fall-risk, boosted by fear of falling.

**Clinical Trial Registration:** www.residentialclinics.gov.br, identifier: RBR-35xhj5.

## Introduction

The study of falls and their predictors amongst the elderly has become increasingly important as the consequences of these events lead to traumatic repercussions both physically and psychologically, contributing to changes in mobility and leading to mortality (1, 2). When it does not reach fatal consequences, the fall may bring reduction in both mobility and social participation due to fear, a condition called “post-fall syndrome” (3). As a result, a vicious and dangerous cycle is generated because fear significantly reduces physical activities to protect itself from the conditions that can cause the fall, but this condition leads to increased comorbidities that promote an increased risk of falls (4).

The fear of falling (FOF) is reported as one of the main predictors of falls (5–8). It is as important as impaired balance (9) or, even more important than the history of falls, since it is present even in the older adults who never fell (10). Applying cognitive theory in the study of fear, it is observed that the subject, when exposed to challenging situations, should not only present necessary skills, but believe that they can deal with them (11). Thus, the study of FOF is based on the concept of self-efficacy, establishing itself by the combination of abilities, motivation, and confidence (12).

As well as fall-risk, the fear of falling is a multidimensional phenomenon, influenced by physical, psychological, social and functional factors (3). Several characteristics are related to fear: being female (13–15), older (15), having poor perception of health (14), higher dependence in the activities of daily living (14, 15), reduced muscle strength (15, 16), impaired balance (14, 15, 17) and previous history of falls (14–16).

In dynamic activities the fear of falling is presented with the adoption of a cautious gait pattern, with significant reductions in different parameters, in particular the walking speed (16, 18, 19). The spatiotemporal and kinematic parameters have been reported as critical clinical tools for assessing the risk of falls in the older adults (20–23). However, the lack of investigations of the extrinsic interferences in gait behavior in older adults, makes the ability of these parameters to predict falls in the elderly population not be clear (24).

The mechanisms underlying the relationship between FOF and falling are not well known, and little attention has been given to the study of their relationship creating a research gap (25). Investigations on gait pattern changes during adverse situations, using obstacles, floor interferences, provoking slippage or footwear modifications have already been done (26–29), however no relationship between gait adaptations and FOF were found. One of the possible methods to investigate the influence of FOF without exposing the participant to unnecessary risks is the application of the “affordances” theory. Proposed in 1979 (30, 31), the “affordances” theory has been applied to neuromotor behavior (32), determining that a visual object can potentiate motor responses even in the absence of actual intention or execution of the task proposed by this object (perception drives action) (33). In some behavioral experiments applying the theory, studies show that they have shown that actions can be enhanced after seeing an image of an object that offer some kind of action, but do not do it (34). Findings provide additional support for the notion that the physical properties of objects automatically activate specific motor codes, but also demonstrate that such influence is rapid and relatively short (32).

Differently from previous studies investigating gait modifications arising from motor perturbations (35), the main aim of this study is to investigate gait kinematic changes in the elderly women exposed to a fictional disturbing factor, using Theory of Affordances. Our secondary aims are: to analyze the gait pattern after disturbance in the elderly women stratified by fall-history and fear of falling; investigating whether demographic factors, cognition and muscle strength can be associated with gait modifications.

## Materials and Methods

### Study Design

This controlled, non-randomized, clinical trial was approved by the Research Ethics Committee of the University of Brasília-College of Ceilândia, decision number 2.109.807 and was conducted in accordance with the Declaration of Helsinki (36). The study was registered in the Brazilian Registry of Clinical Trials (ReBEC) with the code RBR-35xhj5, receiving the number U1111-1222-4514 from the International Clinical Trials Registry Platform (ICTRP) and followed the recommendations of CONSORT (Consolidated Standards of Reporting Trials) (37).

### Participants

Participants were invited to participate in the study which was conducted at the Dr. Cláudio de Almeida Borges Movement Laboratory of the State University of Goiás, Goiânia, Brazil, from August to November 2017. The inclusion criteria were: (i) woman; (ii) age 65 or over; (iii) independent walking without aids; (iv) body mass index (BMI) < 30 kg/m^2^ (38); (v) preserved cognition (Mini-Mental State Examination >24) (39) and >14 points considering the participants the educational level, with illiterate participants (40); (vi) declare that she has not ingested alcoholic beverages within 24 h prior to data collection; (vii) has no prior contact with any gait analysis lab or equipment. The exclusion criteria were: (i) previous surgeries in the lower limbs, pelvis or spine; (ii) have medical diagnosis of rheumatoid arthritis, neuromuscular or neurodegenerative disease, including diabetes mellitus; (iii) visual impairment; (iv) inclusion in other trials. All eligible participants were informed and signed the consent form.

The sample size was determined using G^*^Power software 3.1.9.2 (Franz Faul, Universitat Kiel, Germany) (41), considering one-way variance (ANOVA) of the GPS (Overall) index obtained after perturbation. Thus, the sample required to detect a significant and clinically relevant difference from FOF exposure was *N* = 40 (*n* = 10, per group), effect size (ω^2^) = 0.82, *p* < 0.05, power 0.99.

### Experimental Setup

The participants answered a fall-history questionnaire reporting fall events over the last 12 months. A fall was defined as an “unexpected event in which the participant finds herself on a lower level” (42). To assess FOF, we used the Falls Efficacy Scale-International in its validated version to the Brazilian population (43). It provides information on level of concern about falls for a range of daily activities through 16 questions, each scoring from 1 (not concerned at all) to 4 (very concerned). The final score ranges from 16 to 64. Scores under 27 reveal low concern and over that point, high concern (44). Participants were then assigned into four groups: Faller with low FOF (Fall-LFOF), faller with high FOF (Fall-HFOF), non-faller with low FOF (NonFall-LFOF) and non-faller with high FOF (NonFall-HFOF).

### Data Collection

To perform 3D gait analysis we used the Vicon System (Vicon Motion Systems Ltd®, Oxford Metrics Group, Oxford, UK) and the Conventional Gait Model for biomechanical modeling. All data were sampled at 120 Hz and processed using a fourth-order Butterwoth filter with 10 Hz cut-off frequency (45).

Each volunteer walked barefoot over a 9 meters walkway at a self-selected speed. Two fixed squared metal plates were added at midpoint over the course (Figure 1 in Supplement A). Prior to data collection they went through the walkway five times for familiarization.

**Figure 1:**
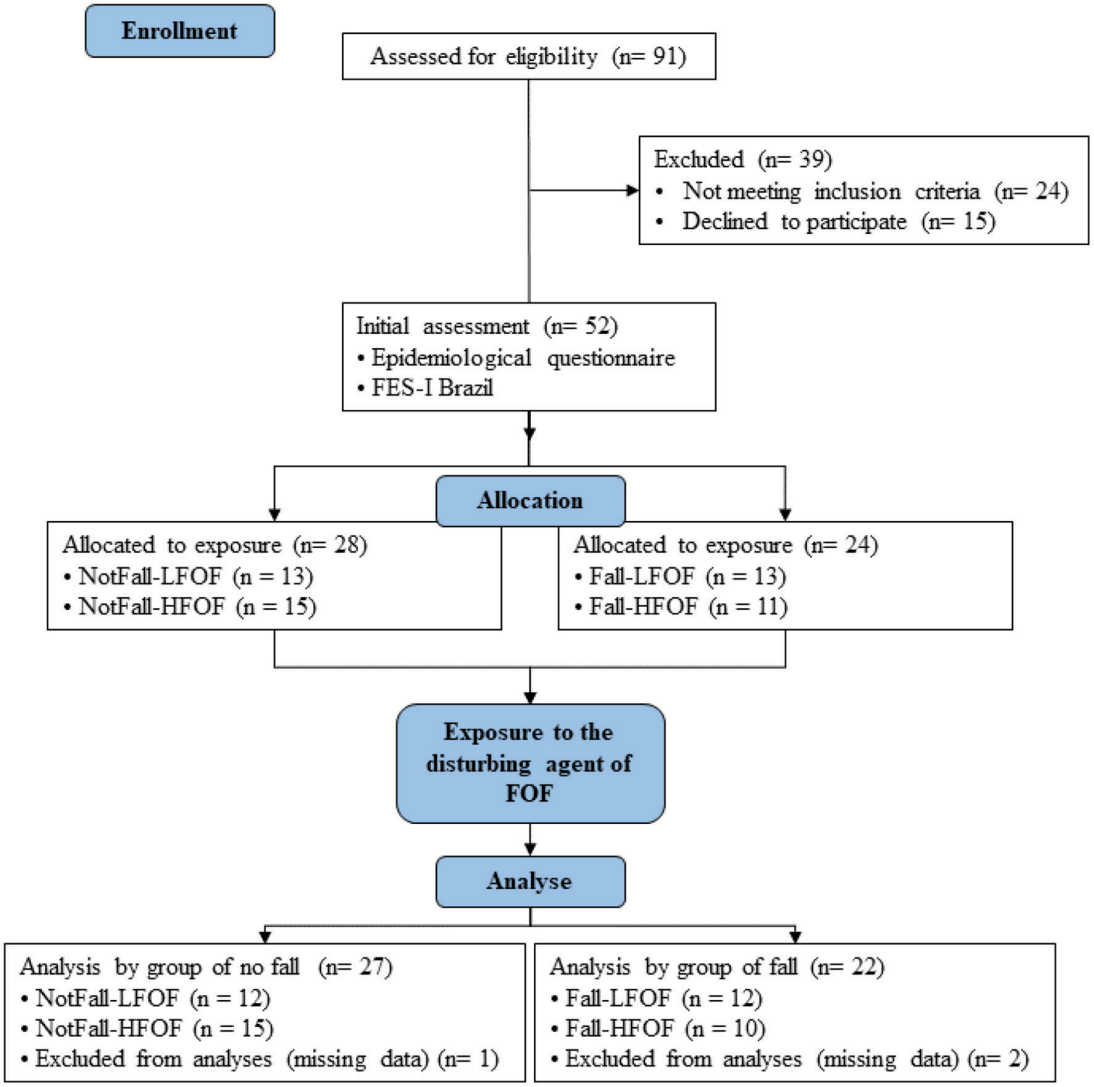
Study flowchart.

After 5 undisturbed gait trials, the participants were warned that the fixed squared objects on the floor could strongly vibrate or deliver electrical discharges when stepped over, introducing a fictional disturbing factor (FDF) to create FOF. Only 2 more trials were collected after introducing FDF to keep participants from getting used to the fictional stimuli (32).

Maximum voluntary isometric contraction (MVIC) was assessed using a manual dynamometer (Laffayete Instrument® Evaluation, Ohio, USA) testing the following muscle groups: hip flexors, extensors, adductors and abductors; knee extensors and flexors; ankle dorsiflexors and plantarflexors. Each muscle group was tested 3 times for 5 s with 1-min rest in between. The highest value was used for analysis. The subject was positioned as standardized by others (46). Right and left side's recordings were averaged and normalized by BMI (47). MVIC was collected after gait trials to avoid muscular fatigue effect on gait pattern (48).

### Data Processing

All kinematic data were normalized by the gait cycle using 51 time-normalized samples for each stride. The averaged gait data pre and post-FDF for right and left sides and for each of the four study groups were analyzed.

The Gait Profile Score (GPS) were used to calculate the quality of gait kinematic parameters (49). The GPS consists of nine gait variable scores (GVS) representing the pelvis, hip, knee and ankle kinematic data, presented in degrees. GVS scores can indicate which joint movement abnormalities tend to contribute to a high (worse) GPS. Both scores were calculated as recommended by Baker and colleagues (49, 50). In this study, the normal group to calculate GPS consisted of 15 women adults with an average age of 24.8 ± 6.8 years old. The data set contained five trials from each subject, resulting in 75 cycles on each lower limb.

### Confounders

Confounders such as age, gender, body weight, body height, BMI were controlled, as well as others that are known to be associated with both fall and FOF repercussions: cognitive level (14); muscle strength (15, 16); and historical fall (14–16).

### Statistical Analysis

Statistical analysis was performed with SPSS Statistics version 23.0 (IBM, Chicago, USA). To assess the normal distribution the Shapiro-Wilk test was used. Tukey's *post-hoc* analysis of variance (ANOVA) was used to analyze the differences between the four groups in the two moments of the study, considering the effect size for the variance (ω) and *post-hoc* comparison. The effect of exposure to FOF agent was analyzed by applying the paired *t*-test, considering the effect size. In order to evaluate the relationship between discriminative variables, muscle strength and temporal space parameters with GPS, the Pearson product correlation was calculated. Correlation of *r* ≤ 0.3 was considered “weak,” 0.31 to 0.69 “substantial” and ≥ 0.7 “strong” (51). The standard level of significance used was 0.05.

## Results

### Demographic Characteristics

During the study period, 91 senior women were eligible to participate in the study. Of these, 52 signed the consent form and participated in the previous evaluation for allocation of the groups. At the end of the study, however, 49 participants remained, being NonFall-LFOF (*n* = 12); NonFall-HFOF (*n* = 15); Fall-LFOF (*n* = 12); FallHFOF (*n* = 10), according to the conditions presented in the flowchart (Figure 1). The results discard the absence of interference of confounders such as age, weight, BMI, as homogeneity was found between groups (*p* < 0.05; Table 1).

**Table 1:** Descriptive and comparative data between NonFall-LFOF, NonFall-HFOF, Fall-LFOF and Fall-HFOF groups.

		**N**	**Mean**	**Std. deviation**	**Std. error**	**95% Confidence interval**		**F**	***p* (ω)**	**Paired comparison**					
						**Lower bound**	**Upper bound**			**A/B (r)**	**A/C (r)**	**A/D (r)**	**B/C (r)**	**B/D (r)**	**C/D (r)**
Age (years)	NonFall -LFOF	12	72.50	6.04	1.74	68.66	76.34	0.411	0.746 (−0.04)	-	-	-	-	-	-
	NonFall -HFOF	15	72.67	7.59	1.96	68.46	76.87								
	Fall -LFOF	12	70.83	5.59	1.61	67.28	74.38								
	Fall -HFOF	10	73.90	6.56	2.07	69.21	78.59								
	Total	49	72.43	6.44	0.92	70.58	74.28								
Weight (Kg)	NonFall -LFOF	12	61.61	6.37	1.84	57.56	65.66	0.694	0.560 (−0.02)	-	-	-	-	-	-
	NonFall -HFOF	15	58.05	10.03	2.59	52.50	63.61								
	Fall -LFOF	12	60.53	8.73	2.52	54.98	66.07								
	Fall -HFOF	10	63.27	11.62	3.67	54.96	71.58								
	Total	49	60.59	9.23	1.32	57.94	63.24								
Height (meters)	NonFall -LFOF	12	1.55	0.05	0.01	1.52	1.59	0.662	0.580 (−0.02)	-	-	-	-	-	-
	NonFall -HFOF	15	1.54	0.05	0.01	1.51	1.56								
	Fall -LFOF	12	1.56	0.08	0.02	1.51	1.60								
	Fall -HFOF	10	1.53	0.06	0.02	1.48	1.57								
	Total	49	1.54	0.06	0.01	1.53	1.56								
BMI (kg/m^2^)	NonFall -LFOF	12	25.57	2.65	0.77	23.88	27.25	1.006	0.399 (0.00)	-	-	-	-	-	-
	NonFall -HFOF	15	24.67	4.53	1.17	22.16	27.18								
	Fall -LFOF	12	24.91	2.34	0.67	23.42	26.39								
	Fall -HFOF	10	27.04	3.97	1.25	24.20	29.88								
	Total	49	25.43	3.55	0.51	24.41	26.45								
Mine mental (score)	NonFall -LFOF	12	26.50	3.15	0.91	24.50	28.50	1.736	0.173 (0.04)	-	-	-	-	-	-
	NonFall -HFOF	15	26.93	2.49	0.64	25.55	28.31								
	Fall -LFOF	12	25.00	3.19	0.92	22.97	27.03								
	Fall -HFOF	10	27.70	2.87	0.91	25.65	29.75								
	Total	49	26.51	2.98	0.43	25.65	27.37								
FES-I (score)	NonFall -LFOF	12	22.33	3.87	1.12	19.88	24.79	21.810	<0.001 (0.56)	<0.001 (0.77)	0.972 (0.11)	<0.001 (0.77)	<0.001 (0.75)	0.701 (0.19)	<0.001 (0.75)
	NonFall -HFOF	15	34.27	5.87	1.52	31.01	37.52								
	Fall -LFOF	12	23.17	3.74	1.08	20.79	25.54								
	Fall -HFOF	10	32.20	4.49	1.42	28.99	35.41								
	Total	49	28.20	7.09	1.01	26.17	30.24								

*A, NonFall-LFOF; B, NonFall-HFOF; C, Fall-LFOF; D, Fall-HFOF. Comparative analysis performed by ANOVA one way, considering the F ratio, effect size (ω) and significance of α ≤ 0.05. Post Tukey post hoc analysis, considering effect size (r) and significance of α ≤ 0.05*.

### Intergroup Comparison of Gait Parameters and MIVM

The step length, stride length, and walking speed showed significant differences between the groups (*p* < 0.05). However, the paired comparison highlighted the NonFall-HFOF group (*r* > 0.40), with reduced walking speed and shorter length in spatial variables pre-FDF. After FDF, only the stride length was different between groups, being lower in the NonFall-HFOF group (Table 1 in Supplement A).

The GPS was not different between the groups, pre-FDF. Three parameters of GVS (Left Ankle Dor/Plan; Left Hip Int/Ext; Right Hip Int/Ext) presented differences between groups (*p* < 0.05) (Table 2 in Supplement A).

**Table 2:** Comparison of the spatiotemporal parameters between pre and post fictional disturbing factor for each of NonFall-LFOF and NonFall- groups.

		**NonFall-LFOF**							**NonFall-HFOF**						
		**Mean**	**N**	**Std. Deviation**	**Std. Error Mean**	***t***	***r***	***p***	**Mean**	**N**	**Std. Deviation**	**Std. Error Mean**	***t***	***r***	***p***
Cadence (steps/min)	Not exposed	110.62	12	7.83	2.26	1.95	0.50	0.077	107.24	15	12.30	3.18	1.46	0.36	0.167
	Exposed	104.19	12	11.99	3.46				104.64	15	14.52	3.75			
Stride time (s)	Not exposed	1.09	12	0.08	0.02	−1.90	0.50	0.084	1.14	15	0.14	0.03	−2.13	0.49	0.051
	Exposed	1.19	12	0.19	0.06				1.18	15	0.17	0.04			
Opposite foot off (%)	Not exposed	9.60	12	1.83	0.53	−3.87	0.76	0.003	10.97	15	2.92	0.75	−2.26	0.55	0.026
	Exposed	11.81	12	2.26	0.65				14.40	15	6.46	1.67			
Opposite foot contact (%)	Not exposed	50.21	12	0.73	0.21	0.33	0.10	0.745	50.11	15	0.67	0.17	−1.21	0.31	0.244
	Exposed	49.94	12	3.15	0.91				50.98	15	2.55	0.66			
StepTime (s)	Not exposed	0.54	12	0.04	0.01	−1.60	0.44	0.137	0.57	15	0.07	0.02	−1.07	0.28	0.301
	Exposed	0.61	12	0.15	0.04				0.58	15	0.09	0.02			
Single support (s)	Not exposed	0.44	12	0.02	0.01	−0.46	0.14	0.657	0.44	15	0.04	0.01	0.45	0.12	0.663
	Exposed	0.46	12	0.10	0.03				0.43	15	0.07	0.02			
Double support (s)	Not exposed	0.22	12	0.04	0.01	−2.20	0.55	0.050	0.27	15	0.09	0.02	−2.47	0.55	0.027
	Exposed	0.32	12	0.14	0.04				0.35	15	0.18	0.05			
Foot off (%)	Not exposed	61.07	12	1.80	0.52	−2.32	0.57	0.041	62.38	15	3.02	0.78	−2.09	0.51	0.048
	Exposed	63.16	12	2.82	0.81				64.44	15	4.91	1.27			
Stride length (m)	Not exposed	1.14	12	0.09	0.03	3.05	0.68	0.011	0.97	15	0.19	0.05	3.39	0.67	0.004
	Exposed	1.02	12	0.13	0.04				0.84	15	0.27	0.07			
Step length (m)	Not exposed	0.57	12	0.05	0.01	1.54	0.42	0.153	0.48	15	0.09	0.02	2.25	0.52	0.041
	Exposed	0.53	12	0.09	0.03				0.43	15	0.15	0.04			
Walking speed (m/s)	Not exposed	1.05	12	0.14	0.04	3.295	0.70	0.007	0.87	15	0.22	0.06	3.83	0.72	0.002
	Exposed	0.88	12	0.17	0.05				0.74	15	0.29	0.08			

*Comparative analysis performed by paired t-test, considering the equation, effect size (r) and significance of α ≤ 0.05*.

After the FOF perturbation, the GPS (Left) and GPS (Overall) presented differences with significant effect between the groups, and the *post hoc* comparison showed only difference between NonFall-HFOF / Fall-LFOF groups, where again NonFall-HFOF presented higher degree of variation in both parameters (Table 2 in Supplement A).

The difference in MVIC was observed only in the muscular group of the plantiflexors between study groups [*F*_(3.45 = 2.809)_, p = 0.050, ω = 0.13], but did not present significant values in the comparison between the pairs (Table 3 in Supplement A).

**Table 3:** Comparison of the spatiotemporal parameters between pre and post fictional disturbing factor for each of Fall-LFOF and Fall-HFOF groups.

		**Fall-LFOF**							**Fall-HFOF**						
		**Mean**	**N**	**Std. Deviation**	**Std. Error Mean**	***t***	***r***	***p***	**Mean**	**N**	**Std. Deviation**	**Std. Error Mean**	***t***	***r***	***p***
Cadence (steps/min)	Not exposed	111.61	12	8.51	2.46	0.89	0.26	0.394	110.28	10	10.46	3.31	1.15	0.36	0.280
	Exposed	110.01	12	9.76	2.82				105.73	10	12.93	4.09			
Stride time (s)	Not exposed	1.08	12	0.09	0.03	−0.89	0.26	0.394	1.10	10	0.11	0.04	−1.05	0.33	0.322
	Exposed	1.10	12	0.10	0.03				1.16	10	0.18	0.06			
Opposite foot off (%)	Not exposed	9.27	12	2.07	0.60	−1.89	0.49	0.086	10.11	10	1.82	0.58	−1.65	0.48	0.134
	Exposed	10.82	12	2.69	0.78				11.69	10	3.13	0.99			
Opposite foot contact (%)	Not exposed	49.92	12	0.63	0.18	0.54	0.16	0.598	50.07	10	0.67	0.21	−0.74	0.24	0.477
	Exposed	49.70	12	1.43	0.41				50.50	10	1.66	0.53			
StepTime (s)	Not exposed	0.54	12	0.04	0.01	−1.06	0.30	0.312	0.55	10	0.05	0.02	−1.13	0.35	0.287
	Exposed	0.55	12	0.05	0.01				0.57	10	0.08	0.02			
Single support (s)	Not exposed	0.44	12	0.03	0.01	1.03	0.30	0.324	0.43	10	0.03	0.01	−0.48	0.16	0.644
	Exposed	0.43	12	0.05	0.01				0.44	10	0.05	0.02			
Double support (s)	Not exposed	0.22	12	0.06	0.02	−1.70	0.46	0.117	0.26	10	0.07	0.02	−1.36	0.41	0.207
	Exposed	0.25	12	0.07	0.02				0.30	10	0.11	0.03			
Foot off (%)	Not exposed	60.59	12	2.44	0.70	−1.55	0.42	0.149	62.65	10	2.28	0.72	−1.40	0.42	0.195
	Exposed	61.71	12	2.87	0.83				63.69	10	2.65	0.84			
Stride length (m)	Not exposed	1.12	12	0.11	0.03	3.09	0.68	0.010	1.04	10	0.07	0.02	3.17	0.73	0.011
	Exposed	1.04	12	0.16	0.05				0.95	10	0.11	0.03			
Step length (m)	Not exposed	0.56	12	0.06	0.02	3.46	0.72	0.005	0.52	10	0.04	0.01	2.92	0.70	0.017
	Exposed	0.52	12	0.08	0.02				0.48	10	0.05	0.02			
Walking speed (m/s)	Not exposed	1.05	12	0.15	0.04	3.54	0.73	0.005	0.96	10	0.15	0.05	2.70	0.67	0.024
	Exposed	0.95	12	0.18	0.05				0.84	10	0.16	0.05			

*Comparative analysis performed by paired t-test, considering the equation, effect size (r) and significance of α ≤ 0.05*.

### Intra-group Comparison of pre and Post-exposure Gait Parameters

After the FDF the modifications of the spatiotemporal parameters were similar between NotFall-LFOF and NotFall-HFOF groups. The opposit foot off and the foot off were late, there was increase of the double support, and reductions were observed in the stride length, walking speed, and the step length reduced only in the NotFall-HFOF group (*p* < 0.05; Table 2). The Fall-LFOF and Fall-HFOF groups presented reduction of the same variables, being the stride length, step length and walking speed (*p* < 0.05; Table 3).

The parameters of the GPS (Left, Right and Overall) did not increase after FDF only in the Fall-HFOF group, however this group already had GPS higher than the other pre-FDF groups (Tables 4, 5). The GVS data show that pre-FDF in all groups the major contributing joints in the GPS range were hip and knee. After the FDF, these joints increased their variations in all groups, remaining as the main responsible for the GPS modification (Tables 4, 5).

**Table 4:** Comparison of GPS and GVS parameters between pre and post fictional disturbing factor for each of NonFall-LFOF and NonFall-HFOF, groups.

		**NonFall-LFOF**							**NonFall-HFOF**						
		**Mean**	**N**	**Std. Deviation**	**Std. Error Mean**	***t***	***r***	***p***	**Mean**	**N**	**Std. Deviation**	**Std. Error Mean**	***t***	***r***	***p***
**GPS (DEGREE)**															
Left	Not exposed	7.22	12	2.01	0.58	−4.49	0.80	0.001	8.52	15	2.41	0.62	−5.21	0.81	<0.001
	Exposed	8.88	12	1.51	0.44				10.49	15	2.48	0.64			
Right	Not exposed	7.09	12	1.70	0.49	−3.57	0.73	0.004	8.43	15	2.31	0.60	−3.42	0.67	0.004
	Exposed	8.51	12	1.61	0.47				9.95	15	2.49	0.64			
Overall	Not exposed	7.61	12	1.75	0.51	−4.96	0.83	<0.001	8.93	15	2.35	0.61	−5.07	0.80	<0.001
	Exposed	9.33	12	1.29	0.37				10.89	15	2.44	0.63			
**GVS (DEGREE)**															
Pelvis ant/post	Not exposed	3.83	12	3.36	0.97	−0.57	0.17	0.578	6.89	15	5.40	1.40	−0.29	0.08	0.777
	Exposed	4.00	12	3.23	0.93				6.97	15	5.60	1.44			
Left Hip flex/ext	Not exposed	9.30	12	5.34	1.54	−1.49	0.41	0.164	12.30	15	7.77	2.01	−2.55	0.56	0.023
	Exposed	10.28	12	4.06	1.17				13.71	15	7.71	1.99			
Left Knee flex/ext	Not exposed	11.97	12	3.26	0.94	−4.03	0.77	0.002	13.03	15	4.70	1.21	−2.37	0.53	0.033
	Exposed	15.80	12	4.66	1.35				15.01	15	6.62	1.71			
Left Ankle dor/plan	Not exposed	4.88	12	1.58	0.46	−3.78	0.75	0.003	7.28	15	2.16	0.56	−1.21	0.31	0.245
	Exposed	6.64	12	1.31	0.38				7.73	15	2.30	0.59			
Pelvic up/dn	Not exposed	2.29	12	0.53	0.15	−2.56	0.61	0.027	3.17	15	1.12	0.29	−1.94	0.46	0.073
	Exposed	2.68	12	0.67	0.19				3.56	15	1.27	0.33			
Left Hip add/abd	Not exposed	5.73	12	2.88	0.83	−0.90	0.26	0.385	5.63	15	2.67	0.69	−3.05	0.63	0.099
	Exposed	6.03	12	3.42	0.99				6.05	15	2.41	0.62			
Pelvic int/ext	Not exposed	5.41	12	3.11	0.90	−0.68	0.20	0.510	4.86	15	1.30	0.34	−2.69	0.58	0.018
	Exposed	5.69	12	2.19	0.63				5.56	15	1.19	0.31			
Left Hip int/ext	Not exposed	5.72	12	5.18	1.49	−2.28	0.57	0.044	6.35	15	0.67	0.17	−6.38	0.86	<0.001
	Exposed	10.38	12	3.73	1.08				14.61	15	5.20	1.34			
Left Foot int/ext	Not exposed	6.33	12	2.43	0.70	−0.48	0.14	0.640	6.75	15	3.43	0.88	−1.32	0.33	0.209
	Exposed	6.69	12	3.13	0.90				7.17	15	3.47	0.90			
Right Hip flex/ext	Not exposed	8.52	12	4.69	1.35	−0.47	0.14	0.646	11.32	15	5.96	1.54	−1.81	0.44	0.092
	Exposed	8.91	12	4.47	1.29				12.93	15	6.06	1.57			
Right Knee flex/ext	Not exposed	9.53	12	3.70	1.07	−2.86	0.65	0.016	13.28	15	4.59	1.19	−3.47	0.68	0.004
	Exposed	12.59	12	4.80	1.38				16.38	15	5.06	1.31			
Right Ankle dor/plan	Not exposed	5.51	12	1.45	0.42	−3.50	0.73	0.005	6.61	15	2.47	0.64	−3.59	0.69	0.003
	Exposed	7.01	12	1.70	0.49				7.90	15	3.04	0.79			
Right Hip add/abd	Not exposed	5.15	12	2.17	0.63	−3.37	0.71	0.006	6.62	15	2.73	0.70	−1.71	0.42	0.110
	Exposed	6.06	12	2.21	0.64				7.10	15	2.64	0.68			
Right Hip int/ext	Not exposed	6.57	12	4.67	1.35	−3.06	0.68	0.011	7.97	15	3.35	0.86	−5.00	0.80	<0.001
	Exposed	11.89	12	3.18	0.92				12.18	15	3.42	0.88			
Right Foot int/ext	Not exposed	8.24	12	4.27	1.23	−0.10	0.03	0.924	6.02	15	2.57	0.66	0.41	0.11	0.687
	Exposed	8.28	12	3.91	1.13				5.85	15	1.70	0.44			

*Comparative analysis performed by paired t-test, considering the equation, effect size (r) and significance of α ≤ 0.05*.

**Table 5:** Comparison of GPS and GVS parameters between pre and post fictional disturbing factor for each of Fall-LFOF and Fall-HFOF, groups.

		**Fall-LFOF**							**Fall-HFOF**						
		**Mean**	**N**	**Std. Deviation**	**Std. Error Mean**	***t***	***r***	***p***	**Mean**	**N**	**Std. Deviation**	**Std. Error Mean**	***t***	***r***	***p***
**GPS (DEGREE)**															
Left	Not exposed	7.47	12	1.34	0.39	−3.10	0.68	0.010	8.74	10	1.01	0.32	−1.29	0.40	0.228
	Exposed	8.46	12	1.62	0.47				9.15	10	1.13	0.36			
Right	Not exposed	7.25	12	1.76	0.51	−2.95	0.66	0.013	8.68	10	1.49	0.47	−1.01	0.32	0.339
	Exposed	8.46	12	2.14	0.62				9.18	10	2.05	0.65			
Overall	Not exposed	7.84	12	1.30	0.38	−3.42	0.72	0.006	9.31	10	1.07	0.34	−1.43	0.43	0.185
	Exposed	9.07	12	1.65	0.48				9.86	10	1.48	0.47			
**GVS (DEGREE)**															
Pelvis ant/post	Not exposed	4.44	12	4.09	1.18	−1.33	0.37	0.210	4.46	10	3.33	1.05	−0.14	0.05	0.890
	Exposed	4.84	12	4.46	1.29				4.54	10	2.93	0.93			
Left Hip flex/ext	Not exposed	7.93	12	3.50	1.01	−0.90	0.26	0.389	10.62	10	4.01	1.27	−1.28	0.39	0.233
	Exposed	8.48	12	3.63	1.05				11.39	10	3.90	1.23			
Left Knee flex/ext	Not exposed	12.85	12	3.92	1.13	−1.45	0.40	0.175	13.61	10	3.46	1.10	−2.04	0.56	0.047
	Exposed	14.15	12	4.83	1.39				15.42	10	2.95	0.93			
Left Ankle dor/plan	Not exposed	5.38	12	1.42	0.41	−2.85	0.65	0.016	6.53	10	2.75	0.87	−1.94	0.54	0.085
	Exposed	6.73	12	2.14	0.62				7.52	10	3.08	0.97			
Pelvic up/dn	Not exposed	2.66	12	1.32	0.38	0.17	0.05	0.868	3.50	10	2.33	0.74	0.26	0.09	0.800
	Exposed	2.62	12	1.36	0.39				3.35	10	1.80	0.57			
Left Hip add/abd	Not exposed	4.43	12	2.03	0.59	−5.52	0.846	0.076	5.45	10	1.97	0.62	−4.88	0.85	0.001
	Exposed	5.31	12	2.02	0.58				6.23	10	2.32	0.73			
Pelvic int/ext	Not exposed	4.55	12	1.98	0.57	−1.68	0.45	0.120	5.09	10	2.40	0.76	0.39	0.13	0.708
	Exposed	5.07	12	1.90	0.55				4.86	10	1.62	0.51			
Left Hip int/ext	Not exposed	8.68	12	2.27	0.66	−3.63	0.74	0.004	13.66	10	0.13	0.04	0.87	0.28	0.405
	Exposed	11.68	12	3.36	0.97				12.59	10	3.79	1.20			
Left Foot int/ext	Not exposed	7.26	12	3.09	0.89	2.02	0.52	0.068	4.60	10	2.37	0.75	−0.38	0.13	0.713
	Exposed	6.53	12	3.19	0.92				4.70	10	1.88	0.59			
Right Hip flex/ext	Not exposed	8.52	12	5.23	1.51	−2.39	0.58	0.036	9.36	10	3.47	1.10	−1.39	0.42	0.197
	Exposed	9.36	12	5.37	1.55				10.16	10	3.52	1.11			
Right Knee flex/ext	Not exposed	11.42	12	3.99	1.15	−1.51	0.42	0.158	12.92	10	4.66	1.47	−2.25	0.60	0.041
	Exposed	12.69	12	4.36	1.26				15.13	10	5.73	1.81			
Right Ankle dor/plan	Not exposed	5.28	12	1.61	0.46	−2.99	0.67	0.012	7.11	10	1.65	0.52	−1.07	0.34	0.310
	Exposed	6.73	12	1.81	0.52				7.99	10	2.95	0.93			
Right Hip add/abd	Not exposed	5.39	12	2.45	0.71	−2.88	0.66	0.015	5.50	10	2.44	0.77	−0.76	0.25	0.464
	Exposed	5.88	12	2.64	0.76				5.91	10	1.79	0.57			
Right Hip int/ext	Not exposed	8.51	12	1.64	0.47	−3.27	0.70	0.007	13.75	10	0.32	0.10	0.63	0.21	0.545
	Exposed	12.14	12	3.34	0.96				13.19	10	2.93	0.93			
Right Foot int/ext	Not exposed	6.18	12	3.50	1.01	−1.29	0.36	0.223	6.71	10	3.17	1.00	−0.66	0.22	0.525
	Exposed	7.07	12	3.83	1.10				7.02	10	3.88	1.23			

*Comparative analysis performed by paired -test, considering the equation, effect size (r) and significance of α ≤ 0.05*.

### Intra-group Correlations Between Confounding Variables and Gait Parameters Pre and Post-exposure to the FOF Agent

The correlation between muscle strength and GPS, showed that the reduction of muscle strength of hip extensors and flexors, and knee flexors contributes to worsening post-FDF gait quality in the NotFall-LFOF group (*r* > 0.6; *p* < 0.05). A similar relationship was found for knee flexors in the Fall-LFOF group (Supplement B).

In the spatiotemporal parameters, correlations were found with the variation of the GPS with the late opposit foot off, late foot off, and increase of the double support. In the NotFall-HFOF group these correlations were observed pre-FDF, and post-FDF increased (*r* > 0.6; *p* < 0.05). Already in the Fall-LFOF group this correlation appeared only post-FDF. And in the Fall-HFOF group, pre-and post-FDF, the correlation was found only between the increase of the double support and the late foot off (Supplement B).

## Discussion

This study aimed to examine the gait pattern adopted by older women exposed to FOF perturbation, and how this factor affects faller and non-faller, with low and high FOF, reflecting in worsening or not the spatiotemporal parameters, GPS and GVS. Significant results pointed to different gait patterns pre and post-FDF. After exposure, all groups presented a reduction in stride length, step length and walking speed, assuming a “cautious” pattern.

Results showed that non-fallers with high FOF change their gait pattern to a cautious gait more than fallers do. The decrease of spatiotemporal variables contrasts with studies that highlight more significant decreases amongst elderly fallers (52, 53). The fact that changes were higher in the presence of FOF than with history of falls agrees with another investigation (48). The introduction of a FOF perturbation during gait resulted in a reduction of the stride length, more significantly in subjects with FOF without fall-history. However, the caution observed by the modifications of other spatiotemporal parameters was similar between groups. This same behavior may be due to declines in the attention process in dynamic or disturbed motor activities, generated by the aging process, where motor slowing are required so that attention on the proposed object remains high (52).

Investigation of FOF effect on the nervous system shows that there is no relation with cognitive decline (54), so the understanding generated by the information offered in the experiment does not differentiate the participants by cognitive interference. The FOF tends to generate an illusory motor image in these older adults, where they feel more agile (Time Up and Go test) than they actually are (25). Thus, assuming a motor pattern that does not match the necessary modifications, not preparing for a motor perturbation that they may suffer.

The sum of the two clinical conditions “to have FOF” and “to have fallen,” together potentiate a gait pattern with opposite and unconscious protection effect. This fact may justify how history of fall and FOF are great predictors of falls (44) since they lead to a pattern of locomotion that predisposes to fall and does not avoid it. The same is observed by other studies that point to the increase in the risk of falls due to the slowing of walking speed (55–57), increased double support (24, 55) and stride length shortening (24). Also, falls prevention is linked to clinical interventions that seek to increase walking speed (58).

The use of “caution,” potentiated by FOF, causes gait perturbation, with changes in the kinematic parameters (59), and the slowing of locomotion will corroborate the loss of gait quality (60). These same adaptations and consequent worsening of gait quality observed with higher intensity in our sample of elderly women who presented high FOF and no fall history. Compensations in kinematics to avoid the reduction of gait quality are noted by all groups, where they prolong the timing of opposite foot off (61), and foot off (62), occurring due to weight transfer and foot release being the less stable periods of the gait cycle (61, 62).

The adjustments to try to maintain the gait quality seem to be inefficient since it was observed that the larger joints such as hip and knee are the greatest responsible for gait abnormality in this sample. A meta-analysis shows that to maintain gait quality with advancing age the hip increases its contribution, but they do not explain to what extent this increase in contribution is good or not to reduce the risk of falls (63). Our data show that the joints of the hip and knee were in all groups the joints that contributed the most to the variation of normal gait measured by the GPS, after perturbation. Studies have indicated that these joints are the ones with the most variations in segmental coordination in periods of gait instability (62–64). Moreover, the motor variation of these joints is more considerable in the presence of FOF (65, 66) and intensified by the need for an organization to an unexpected perturbation or obstacle during walking (65).

Because of that, the strategy to reduce the spatiotemporal parameters of gait is an attempt to promote greater time adjustment, in the dynamic segmental coordination, promoting caution, when going through the disturbing factor. In situations where older adults need to maintain a gait pattern and ensure attention to a stimulus, they end up prioritizing the maintenance of a “cautious” gait pattern in order to reduce the risk of falling (67). It is known that in older adults with fall-risk, gait adaptability in situations that demand attention and adjustment is weakened, and the lack of adaptability increases the risk of falling (68), seek in “caution,” to reduce them with a slower gait when approaching targets or obstacles to locomotion (68). However, in the presence of FOF, the adjustments in gait pattern predispose an increase in the risk of falling and do not have the expected protective effect (24, 67, 69), worsening the quality of gait.

FOF produces anxiety in an attempt to predict the effects of a threatening stimuli that can compromise a task, leading to a memory block of usual motor tasks (70, 71), causing them to adopt a more energetic dynamic posture to try to avoid the loss of balance during threatening situations (18, 19). However, this changes compromise performance in dynamic and demanding functional tasks such as walking, leading to the inadequate acquisition of sensory information necessary to plan and execute postural adjustments in these threatening situations (70). When a target is given or alerted to a stimulus evoking FOF, the older person attempts to focus on the target visually, but when close to it, tends to look away from the target, resulting in worse accuracy to hit the target (72). In the anticipated state that the anxiety generated by the FOF promotes, it increases the risk of falling because it produces a step and an inaccurate displacement (70, 71).

Our findings on the influence of confounders on the interpretation of the effects obtained by the exposition to the disturbing factor highlighted that only the muscular strength of large muscle groups acting on the large joints such as hip and knee presented interferences. This relationship was only observed in those who fell and did not fall with low FOF, corroborating that there is no association between muscle strength and FOF (48). However, exposure to a perturbation of fall showed that the needs of gait adjustments is not conditioned to muscle strength. Thus, we pointed out that the FOF contributes more than fall-history, cognitive level and muscle strength, on the modifications of walking parameters after exposure to a fear agent. Our findings agree with another investigation (73) showing that fall-risk increases only when there are high FOF and poor gait quality.

In the past, the combination of motor skills, motivation, and trust was the most important concept of self-efficacy (11, 12). The subject needs to overcome the FOF in challenging situations, promoting adjustment skills, but also believing that he or she can cope with them (74, 75). It is reasonable to hypothesize that interventions to fall-prevention need to incorporate conditions beyond what is observed in the musculoskeletal system and its functions. The complexity of this is what should move future research addressing the relationship between structure/function of the body and psychological factors.

The findings of this study should also be regarded with some limitations. First, this study was limited by its small sample size, although we followed the values indicated in the sample calculation and considered the homogeneity of demographic variables in the study of aging. A second limitation is that this study was restricted to a group of elderly women, and the findings may differ from elderly men. What is emphasized here is that in the future more external relations may be incorporated in studies of the motor modifications of the elderly population, and thus contributing to prevention and reduction of the risk of falling, with a greater understanding of its complexity and better interpretation for the clinical practice.

## Ethics Statement

This controlled, non-randomized, clinical trial was approved by the Research Ethics Committee of the University of Brasília-College of Ceilândia, decision number 2.109.807. The study was registered in the Brazilian Registry of Clinical Trials (ReBEC) with the code RBR-35xhj5, receiving the number U1111-1222-4514 from the International Clinical Trials Registry Platform (ICTRP) and followed the recommendations of CONSORT (Consolidated Standards of Reporting Trials) (37).

## Author Contributions

GB: analysis and interpretation of the data, study concept, wrote the manuscript. DR, AM, and TL: analysis of data, critical revision of the manuscript for important intellectual content. FG and RdM: study concept and design, study supervision, critical revisions of the manuscript for important intellectual content.

### Conflict of Interest Statement

The authors declare that the research was conducted in the absence of any commercial or financial relationships that could be construed as a potential conflict of interest.
