# Supplementary material for: Fear of Falling Contributing to Cautious Gait Pattern in Women Exposed to a Fictional Disturbing Factor: A Non-randomized Clinical Trial
Source: Front Neurol. 2019 Mar 26;10:283. doi: 10.3389/fneur.2019.00283 (PMC6445048; doi:10.3389/fneur.2019.00283)
Supplement: Supplementary file 1 [file Data_Sheet_1.docx]

**Supplement – A**


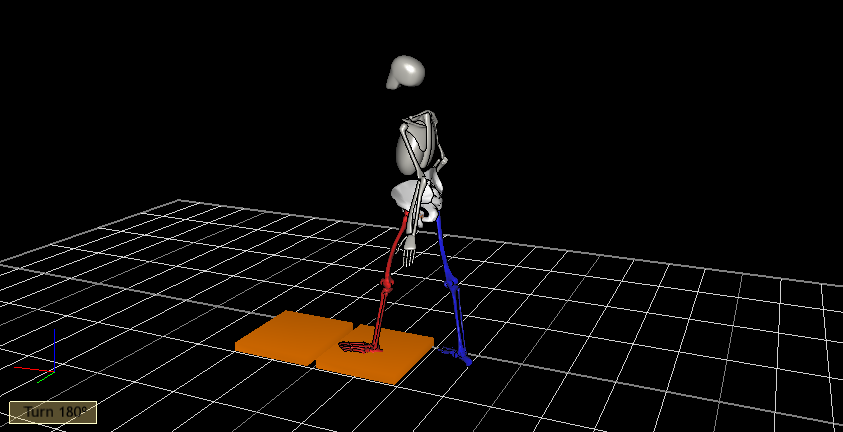


**Figure 1.** Image extracted from the Vicon Polygon software of one of the participating women. In orange are highlighted the two fixed square metal plates used to generate the fictional disturbing factor following the theory of "affordances".


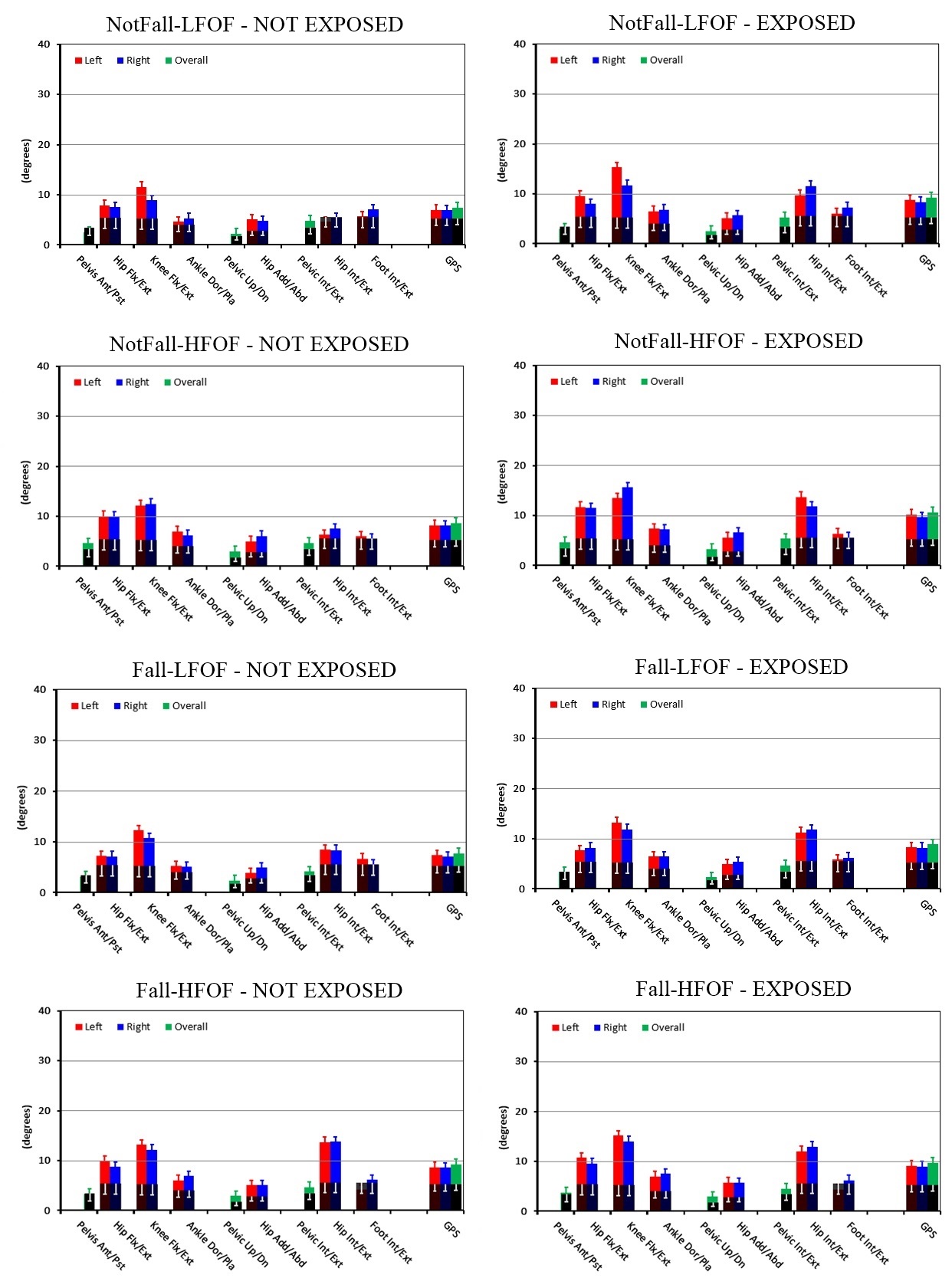


**Figure 2** - GVS / MAP groups of NotFall-LFOF, NotFall-HFOF, Fall-LFOF and Fall-HFOF pre and post fictional disturbing factor

Table 1 - Description and comparison of the spatiotemporal parameters of gait pre and post fictional disturbing factor between NotFall-LFOF, NotFall-HFOF, Fall-LFOF and Fall-HFOF groups.

|  | | N | Mean | Std. Deviation | Std. Error | 95% Confidence Interval | | F | *p* valor (ω²) | *post hoc* | | | | | |
| --- | --- | --- | --- | --- | --- | --- | --- | --- | --- | --- | --- | --- | --- | --- | --- |
|  |  |  |  |  |  | Lower Bound | Upper Bound |  |  | *A/B*  *(r)* | *A/C*  *(r)* | *A/D*  *(r)* | *B/C*  *(r)* | *B/D*  *(r)* | *C/D*  *(r)* |
| *Data Not Exposed* | | | | | | | | | | | | | | | |
| Cadence (steps/min) | NotFall-LFOF | 12 | 110.62 | 7.83 | 2.26 | 105.64 | 115.59 | 0.485 | 0.694  (-0.03) | - | - | - | - | - | - |
|  | NotFall-HFOF | 15 | 107.24 | 12.30 | 3.18 | 100.43 | 114.05 |  |  |  |  |  |  |  |  |
|  | Fall-LFOF | 12 | 111.61 | 8.51 | 2.46 | 106.21 | 117.02 |  |  |  |  |  |  |  |  |
|  | Fall-HFOF | 10 | 110.28 | 10.46 | 3.31 | 102.79 | 117.76 |  |  |  |  |  |  |  |  |
|  | Total | 49 | 109.76 | 9.92 | 1.42 | 106.91 | 112.61 |  |  |  |  |  |  |  |  |
| Stride Time (seconds) | NotFall-LFOF | 12 | 1.09 | 0.08 | 0.02 | 1.04 | 1.14 | 0.628 | 0.601  (-0.02) | - | - | - | - | - | - |
|  | NotFall-HFOF | 15 | 1.14 | 0.14 | 0.03 | 1.06 | 1.21 |  |  |  |  |  |  |  |  |
|  | Fall-LFOF | 12 | 1.08 | 0.09 | 0.03 | 1.03 | 1.14 |  |  |  |  |  |  |  |  |
|  | Fall-HFOF | 10 | 1.10 | 0.11 | 0.04 | 1.02 | 1.18 |  |  |  |  |  |  |  |  |
|  | Total | 49 | 1.11 | 0.11 | 0.02 | 1.07 | 1.14 |  |  |  |  |  |  |  |  |
| Opposite Foot Off (percent) | NotFall-LFOF | 12 | 9.60 | 1.83 | 0.53 | 8.43 | 10.76 | 1.442 | 0.243  (0.03) | - | - | - | - | - | - |
|  | NotFall-HFOF | 15 | 10.97 | 2.92 | 0.75 | 9.35 | 12.59 |  |  |  |  |  |  |  |  |
|  | Fall-LFOF | 12 | 9.27 | 2.07 | 0.60 | 7.96 | 10.59 |  |  |  |  |  |  |  |  |
|  | Fall-HFOF | 10 | 10.11 | 1.82 | 0.58 | 8.81 | 11.42 |  |  |  |  |  |  |  |  |
|  | Total | 49 | 10.04 | 2.31 | 0.33 | 9.38 | 10.71 |  |  |  |  |  |  |  |  |
| Opposite Foot Contact (percent) | NotFall-LFOF | 12 | 50.21 | 0.73 | 0.21 | 49.74 | 50.67 | 0.390 | 0.761  (-0.04) | - | - | - | - | - | - |
|  | NotFall-HFOF | 15 | 50.11 | 0.67 | 0.17 | 49.75 | 50.48 |  |  |  |  |  |  |  |  |
|  | Fall-LFOF | 12 | 49.92 | 0.63 | 0.18 | 49.51 | 50.32 |  |  |  |  |  |  |  |  |
|  | Fall-HFOF | 10 | 50.07 | 0.67 | 0.21 | 49.58 | 50.55 |  |  |  |  |  |  |  |  |
|  | Total | 49 | 50.08 | 0.66 | 0.09 | 49.89 | 50.27 |  |  |  |  |  |  |  |  |
| StepTime (seconds) | NotFall-LFOF | 12 | 0.54 | 0.04 | 0.01 | 0.52 | 0.57 | 0.600 | 0.619  (-0.03) | - | - | - | - | - | - |
|  | NotFall-HFOF | 15 | 0.57 | 0.07 | 0.02 | 0.53 | 0.60 |  |  |  |  |  |  |  |  |
|  | Fall-LFOF | 12 | 0.54 | 0.04 | 0.01 | 0.52 | 0.57 |  |  |  |  |  |  |  |  |
|  | Fall-HFOF | 10 | 0.55 | 0.05 | 0.02 | 0.51 | 0.58 |  |  |  |  |  |  |  |  |
|  | Total | 49 | 0.55 | 0.05 | 0.01 | 0.54 | 0.57 |  |  |  |  |  |  |  |  |
| Single Support (seconds) | NotFall-LFOF | 12 | 0.44 | 0.02 | 0.01 | 0.43 | 0.46 | 0.291 | 0.832  (-0.05) | - | - | - | - | - | - |
|  | NotFall-HFOF | 15 | 0.44 | 0.04 | 0.01 | 0.42 | 0.46 |  |  |  |  |  |  |  |  |
|  | Fall-LFOF | 12 | 0.44 | 0.03 | 0.01 | 0.42 | 0.46 |  |  |  |  |  |  |  |  |
|  | Fall-HFOF | 10 | 0.43 | 0.03 | 0.01 | 0.41 | 0.46 |  |  |  |  |  |  |  |  |
|  | Total | 49 | 0.44 | 0.03 | 0.00 | 0.43 | 0.45 |  |  |  |  |  |  |  |  |
| Double Support (seconds) | NotFall-LFOF | 12 | 0.22 | 0.04 | 0.01 | 0.20 | 0.25 | 1.602 | 0.202  (0.04) | - | - | - | - | - | - |
|  | NotFall-HFOF | 15 | 0.27 | 0.09 | 0.02 | 0.22 | 0.32 |  |  |  |  |  |  |  |  |
|  | Fall-LFOF | 12 | 0.22 | 0.06 | 0.02 | 0.18 | 0.26 |  |  |  |  |  |  |  |  |
|  | Fall-HFOF | 10 | 0.26 | 0.07 | 0.02 | 0.21 | 0.31 |  |  |  |  |  |  |  |  |
|  | Total | 49 | 0.24 | 0.07 | 0.01 | 0.22 | 0.26 |  |  |  |  |  |  |  |  |
| Foot Off (percent) | NotFall-LFOF | 12 | 61.07 | 1.80 | 0.52 | 59.93 | 62.21 | 1.936 | 0.137  (0.05) | - | - | - | - | - | - |
|  | NotFall-HFOF | 15 | 62.38 | 3.02 | 0.78 | 60.71 | 64.05 |  |  |  |  |  |  |  |  |
|  | Fall-LFOF | 12 | 60.59 | 2.44 | 0.70 | 59.04 | 62.14 |  |  |  |  |  |  |  |  |
|  | Fall-HFOF | 10 | 62.65 | 2.28 | 0.72 | 61.02 | 64.28 |  |  |  |  |  |  |  |  |
|  | Total | 49 | 61.67 | 2.55 | 0.36 | 60.94 | 62.41 |  |  |  |  |  |  |  |  |
| Stride Lenght (metres) | NotFall-LFOF | 12 | 1.14 | 0.09 | 0.03 | 1.07 | 1.20 | 5.027 | 0.004  (0.20) | 0.008  (0.50) | 0.997  (0.05) | 0.331  (0.51) | 0.015  (0.46) | 0.508  (0.24) | 0.432  (0.42) |
|  | NotFall-HFOF | 15 | 0.97 | 0.19 | 0.05 | 0.86 | 1.07 |  |  |  |  |  |  |  |  |
|  | Fall-LFOF | 12 | 1.12 | 0.11 | 0.03 | 1.05 | 1.20 |  |  |  |  |  |  |  |  |
|  | Fall-HFOF | 10 | 1.04 | 0.07 | 0.02 | 0.99 | 1.09 |  |  |  |  |  |  |  |  |
|  | Total | 49 | 1.06 | 0.15 | 0.02 | 1.02 | 1.10 |  |  |  |  |  |  |  |  |
| Step Lenght (metres) | NotFall-LFOF | 12 | 0.57 | 0.05 | 0.01 | 0.54 | 0.60 | 5.119 | 0.004  (0.20) | 0.009  (0.50) | 1.000  (0.02) | 0.357  (0.48) | 0.011  (0.48) | 0.492  (0.25) | 0.396  (0.42) |
|  | NotFall-HFOF | 15 | 0.48 | 0.09 | 0.02 | 0.43 | 0.53 |  |  |  |  |  |  |  |  |
|  | Fall-LFOF | 12 | 0.56 | 0.06 | 0.02 | 0.53 | 0.60 |  |  |  |  |  |  |  |  |
|  | Fall-HFOF | 10 | 0.52 | 0.04 | 0.01 | 0.49 | 0.55 |  |  |  |  |  |  |  |  |
|  | Total | 49 | 0.53 | 0.07 | 0.01 | 0.51 | 0.55 |  |  |  |  |  |  |  |  |
| Walking Speed (metres per second) | NotFall-LFOF | 12 | 1.05 | 0.14 | 0.04 | 0.96 | 1.13 | 3.378 | 0.026  (0.13) | 0.049  (0.44) | 1.000  (0.01) | 0.628  (0.31) | 0.046  (0.43) | 0.582  (0.23) | 0.612  (0.30) |
|  | NotFall-HFOF | 15 | 0.87 | 0.22 | 0.06 | 0.75 | 0.99 |  |  |  |  |  |  |  |  |
|  | Fall-LFOF | 12 | 1.05 | 0.15 | 0.04 | 0.95 | 1.15 |  |  |  |  |  |  |  |  |
|  | Fall-HFOF | 10 | 0.96 | 0.15 | 0.05 | 0.86 | 1.06 |  |  |  |  |  |  |  |  |
|  | Total | 49 | 0.98 | 0.18 | 0.03 | 0.92 | 1.03 |  |  |  |  |  |  |  |  |
| *Data Exposed* | | | | | | | | | | | | | | | |
| Cadence (steps/min) | NotFall-LFOF | 12 | 104.19 | 11.99 | 3.46 | 96.57 | 111.80 | 0.551 | 0.650  (-0.03) | - | - | - | - | - | - |
|  | NotFall-HFOF | 15 | 104.64 | 14.52 | 3.75 | 96.60 | 112.68 |  |  |  |  |  |  |  |  |
|  | Fall-LFOF | 12 | 110.01 | 9.76 | 2.82 | 103.80 | 116.21 |  |  |  |  |  |  |  |  |
|  | Fall-HFOF | 10 | 105.73 | 12.93 | 4.09 | 96.48 | 114.98 |  |  |  |  |  |  |  |  |
|  | Total | 49 | 106.07 | 12.37 | 1.77 | 102.51 | 109.62 |  |  |  |  |  |  |  |  |
| Stride Time (seconds) | NotFall-LFOF | 12 | 1.19 | 0.19 | 0.06 | 1.07 | 1.31 | 0.732 | 0.538  (-0.02) | - | - | - | - | - | - |
|  | NotFall-HFOF | 15 | 1.18 | 0.17 | 0.04 | 1.08 | 1.28 |  |  |  |  |  |  |  |  |
|  | Fall-LFOF | 12 | 1.10 | 0.10 | 0.03 | 1.04 | 1.16 |  |  |  |  |  |  |  |  |
|  | Fall-HFOF | 10 | 1.16 | 0.18 | 0.06 | 1.03 | 1.29 |  |  |  |  |  |  |  |  |
|  | Total | 49 | 1.16 | 0.16 | 0.02 | 1.11 | 1.21 |  |  |  |  |  |  |  |  |
| Opposite Foot Off (percent) | NotFall-LFOF | 12 | 11.81 | 2.26 | 0.65 | 10.37 | 13.25 | 1.838 | 0.154  (0.05) | - | - | - | - | - | - |
|  | NotFall-HFOF | 15 | 14.40 | 6.46 | 1.67 | 10.83 | 17.98 |  |  |  |  |  |  |  |  |
|  | Fall-LFOF | 12 | 10.82 | 2.69 | 0.78 | 9.11 | 12.53 |  |  |  |  |  |  |  |  |
|  | Fall-HFOF | 10 | 11.69 | 3.13 | 0.99 | 9.45 | 13.93 |  |  |  |  |  |  |  |  |
|  | Total | 49 | 12.34 | 4.35 | 0.62 | 11.09 | 13.59 |  |  |  |  |  |  |  |  |
| Opposite Foot Contact (percent) | NotFall-LFOF | 12 | 49.94 | 3.15 | 0.91 | 47.94 | 51.94 | 0.807 | 0.496  (-0.01) | - | - | - | - | - | - |
|  | NotFall-HFOF | 15 | 50.98 | 2.55 | 0.66 | 49.57 | 52.40 |  |  |  |  |  |  |  |  |
|  | Fall-LFOF | 12 | 49.70 | 1.43 | 0.41 | 48.79 | 50.60 |  |  |  |  |  |  |  |  |
|  | Fall-HFOF | 10 | 50.50 | 1.66 | 0.53 | 49.31 | 51.69 |  |  |  |  |  |  |  |  |
|  | Total | 49 | 50.31 | 2.33 | 0.33 | 49.64 | 50.98 |  |  |  |  |  |  |  |  |
| StepTime (seconds) | NotFall-LFOF | 12 | 0.61 | 0.15 | 0.04 | 0.52 | 0.71 | 0.750 | 0.528  (-0.02) | - | - | - | - | - | - |
|  | NotFall-HFOF | 15 | 0.58 | 0.09 | 0.02 | 0.53 | 0.63 |  |  |  |  |  |  |  |  |
|  | Fall-LFOF | 12 | 0.55 | 0.05 | 0.01 | 0.52 | 0.59 |  |  |  |  |  |  |  |  |
|  | Fall-HFOF | 10 | 0.57 | 0.08 | 0.02 | 0.52 | 0.63 |  |  |  |  |  |  |  |  |
|  | Total | 49 | 0.58 | 0.10 | 0.01 | 0.55 | 0.61 |  |  |  |  |  |  |  |  |
| Single Support (seconds) | NotFall-LFOF | 12 | 0.46 | 0.10 | 0.03 | 0.39 | 0.52 | 0.349 | 0.790  (-0.04) | - | - | - | - | - | - |
|  | NotFall-HFOF | 15 | 0.43 | 0.07 | 0.02 | 0.39 | 0.47 |  |  |  |  |  |  |  |  |
|  | Fall-LFOF | 12 | 0.43 | 0.05 | 0.01 | 0.40 | 0.46 |  |  |  |  |  |  |  |  |
|  | Fall-HFOF | 10 | 0.44 | 0.05 | 0.02 | 0.41 | 0.48 |  |  |  |  |  |  |  |  |
|  | Total | 49 | 0.44 | 0.07 | 0.01 | 0.42 | 0.46 |  |  |  |  |  |  |  |  |
| Double Support (seconds) | NotFall-LFOF | 12 | 0.32 | 0.14 | 0.04 | 0.23 | 0.41 | 1.248 | 0.304  (0.01) | - | - | - | - | - | - |
|  | NotFall-HFOF | 15 | 0.35 | 0.18 | 0.05 | 0.25 | 0.45 |  |  |  |  |  |  |  |  |
|  | Fall-LFOF | 12 | 0.25 | 0.07 | 0.02 | 0.21 | 0.29 |  |  |  |  |  |  |  |  |
|  | Fall-HFOF | 10 | 0.30 | 0.11 | 0.03 | 0.22 | 0.37 |  |  |  |  |  |  |  |  |
|  | Total | 49 | 0.31 | 0.14 | 0.02 | 0.27 | 0.35 |  |  |  |  |  |  |  |  |
| Foot Off (percent) | NotFall-LFOF | 12 | 63.16 | 2.82 | 0.81 | 61.37 | 64.95 | 1.343 | 0.272  (0.02) | - | - | - | - | - | - |
|  | NotFall-HFOF | 15 | 64.44 | 4.91 | 1.27 | 61.73 | 67.16 |  |  |  |  |  |  |  |  |
|  | Fall-LFOF | 12 | 61.71 | 2.87 | 0.83 | 59.88 | 63.53 |  |  |  |  |  |  |  |  |
|  | Fall-HFOF | 10 | 63.69 | 2.65 | 0.84 | 61.80 | 65.59 |  |  |  |  |  |  |  |  |
|  | Total | 49 | 63.31 | 3.62 | 0.52 | 62.27 | 64.35 |  |  |  |  |  |  |  |  |
| Stride Lenght (metres) | NotFall-LFOF | 12 | 1.02 | 0.13 | 0.04 | 0.93 | 1.10 | 3.056 | 0.038  (0.11) | 0.091  (0.38) | 0.991  (0.08) | 0.862  (0.27) | 0.044  (0.41) | 0.470  (0.25) | 0.712  (0.31) |
|  | NotFall-HFOF | 15 | 0.84 | 0.27 | 0.07 | 0.69 | 0.99 |  |  |  |  |  |  |  |  |
|  | Fall-LFOF | 12 | 1.04 | 0.16 | 0.05 | 0.94 | 1.14 |  |  |  |  |  |  |  |  |
|  | Fall-HFOF | 10 | 0.95 | 0.11 | 0.03 | 0.88 | 1.03 |  |  |  |  |  |  |  |  |
|  | Total | 49 | 0.95 | 0.20 | 0.03 | 0.90 | 1.01 |  |  |  |  |  |  |  |  |
| Step Lenght (metres) | NotFall-LFOF | 12 | 0.53 | 0.09 | 0.03 | 0.47 | 0.58 | 2.511 | 0.071  (0.08) | - | - | - | - | - | - |
|  | NotFall-HFOF | 15 | 0.43 | 0.15 | 0.04 | 0.35 | 0.51 |  |  |  |  |  |  |  |  |
|  | Fall-LFOF | 12 | 0.52 | 0.08 | 0.02 | 0.47 | 0.57 |  |  |  |  |  |  |  |  |
|  | Fall-HFOF | 10 | 0.48 | 0.05 | 0.02 | 0.44 | 0.52 |  |  |  |  |  |  |  |  |
|  | Total | 49 | 0.49 | 0.11 | 0.02 | 0.46 | 0.52 |  |  |  |  |  |  |  |  |
| Walking Speed (metres per second) | NotFall-LFOF | 12 | 0.88 | 0.17 | 0.05 | 0.77 | 0.99 | 2.334 | 0.087  (0.08) | - | - | - | - | - | - |
|  | NotFall-HFOF | 15 | 0.74 | 0.29 | 0.08 | 0.57 | 0.90 |  |  |  |  |  |  |  |  |
|  | Fall-LFOF | 12 | 0.95 | 0.18 | 0.05 | 0.84 | 1.07 |  |  |  |  |  |  |  |  |
|  | Fall-HFOF | 10 | 0.84 | 0.16 | 0.05 | 0.72 | 0.96 |  |  |  |  |  |  |  |  |
|  | Total | 49 | 0.85 | 0.23 | 0.03 | 0.78 | 0.91 |  |  |  |  |  |  |  |  |

Note: A - NotFall-LFOF; B-NotFall-HFOF; C-Fall-LFOF; D - FallHFOF. Data Not Exposed - data obtained before exposure to the fictional disturbing factor; Data Exposed - Data obtained during exposure to the fictional disturbing factor. Comparative analysis performed by ANOVA one way, considering the F ratio, effect size (ω) and significance of α≤0.05. Post Tukey post hoc analysis, considering effect size (r) and significance of α≤0.05.

**Table 2** - Description and comparison of the GPS and GVS parameters pre and post fictional disturbing factor between NotFall-LFOF, NotFall-HFOF, Fall-LFOF and Fall-HFOF groups.

|  | | N | Mean | Std. Deviation | Std. Error | 95% Confidence Interval | | F | *p* valor (ω²) | *post hoc* | | | | | |
| --- | --- | --- | --- | --- | --- | --- | --- | --- | --- | --- | --- | --- | --- | --- | --- |
|  |  |  |  |  |  | Lower Bound | Upper Bound |  |  | *A/B*  *(r)* | *A/C*  *(r)* | *A/D*  *(r)* | *B/C*  *(r)* | *B/D*  *(r)* | *C/D*  *(r)* |
| ***Data Not Exposed*** |  |  |  |  |  |  |  |  |  |  |  |  |  |  |  |
| GPS (Left) (degree) | NotFall-LFOF | 12 | 7.22 | 2.01 | 0.58 | 5.94 | 8.49 | 1.97 | 0.132  (0.06) | - | - | - | - | - | - |
|  | NotFall-HFOF | 15 | 8.52 | 2.41 | 0.62 | 7.18 | 9.86 |  |  |  |  |  |  |  |  |
|  | Fall-LFOF | 12 | 7.47 | 1.34 | 0.39 | 6.62 | 8.32 |  |  |  |  |  |  |  |  |
|  | Fall-HFOF | 10 | 8.74 | 1.01 | 0.32 | 8.02 | 9.46 |  |  |  |  |  |  |  |  |
|  | Total | 49 | 7.99 | 1.91 | 0.27 | 7.44 | 8.54 |  |  |  |  |  |  |  |  |
| GPS (Right) (degree) | NotFall-LFOF | 12 | 7.09 | 1.70 | 0.49 | 6.01 | 8.17 | 2.16 | 0.106  (0.07) | - | - | - | - | - | - |
|  | NotFall-HFOF | 15 | 8.43 | 2.31 | 0.60 | 7.14 | 9.71 |  |  |  |  |  |  |  |  |
|  | Fall-LFOF | 12 | 7.25 | 1.76 | 0.51 | 6.13 | 8.37 |  |  |  |  |  |  |  |  |
|  | Fall-HFOF | 10 | 8.68 | 1.49 | 0.47 | 7.62 | 9.74 |  |  |  |  |  |  |  |  |
|  | Total | 49 | 7.86 | 1.96 | 0.28 | 7.30 | 8.43 |  |  |  |  |  |  |  |  |
| GPS (Overall) (degree) | NotFall-LFOF | 12 | 7.61 | 1.75 | 0.51 | 6.49 | 8.72 | 2.55 | 0.067  (0.09) | - | - | - | - | - | - |
|  | NotFall-HFOF | 15 | 8.93 | 2.35 | 0.61 | 7.63 | 10.23 |  |  |  |  |  |  |  |  |
|  | Fall-LFOF | 12 | 7.84 | 1.30 | 0.38 | 7.01 | 8.67 |  |  |  |  |  |  |  |  |
|  | Fall-HFOF | 10 | 9.31 | 1.07 | 0.34 | 8.55 | 10.07 |  |  |  |  |  |  |  |  |
|  | Total | 49 | 8.42 | 1.85 | 0.26 | 7.89 | 8.95 |  |  |  |  |  |  |  |  |
| LEFT Pelvis Ant/Pst (degree) | NotFall-LFOF | 12 | 3.83 | 3.36 | 0.97 | 1.69 | 5.96 | 1.41 | 0.254  (0.02) | - | - | - | - | - | - |
|  | NotFall-HFOF | 15 | 6.89 | 5.40 | 1.40 | 3.89 | 9.88 |  |  |  |  |  |  |  |  |
|  | Fall-LFOF | 12 | 4.44 | 4.09 | 1.18 | 1.84 | 7.04 |  |  |  |  |  |  |  |  |
|  | Fall-HFOF | 10 | 4.46 | 3.33 | 1.05 | 2.08 | 6.84 |  |  |  |  |  |  |  |  |
|  | Total | 49 | 5.04 | 4.31 | 0.62 | 3.80 | 6.28 |  |  |  |  |  |  |  |  |
| LEFT Hip Flx/Ext (degree) | NotFall-LFOF | 12 | 9.30 | 5.34 | 1.54 | 5.90 | 12.70 | 1.45 | 0.240  (0.03) | - | - | - | - | - | - |
|  | NotFall-HFOF | 15 | 12.30 | 7.77 | 2.01 | 8.00 | 16.60 |  |  |  |  |  |  |  |  |
|  | Fall-LFOF | 12 | 7.93 | 3.50 | 1.01 | 5.71 | 10.16 |  |  |  |  |  |  |  |  |
|  | Fall-HFOF | 10 | 10.62 | 4.01 | 1.27 | 7.75 | 13.49 |  |  |  |  |  |  |  |  |
|  | Total | 49 | 10.15 | 5.73 | 0.82 | 8.51 | 11.80 |  |  |  |  |  |  |  |  |
| LEFT Knee Flx/Ext (degree) | NotFall-LFOF | 12 | 11.97 | 3.26 | 0.94 | 9.89 | 14.04 | 0.33 | 0.801  (-0.04) | - | - | - | - | - | - |
|  | NotFall-HFOF | 15 | 13.03 | 4.70 | 1.21 | 10.43 | 15.63 |  |  |  |  |  |  |  |  |
|  | Fall-LFOF | 12 | 12.85 | 3.92 | 1.13 | 10.36 | 15.34 |  |  |  |  |  |  |  |  |
|  | Fall-HFOF | 10 | 13.61 | 3.46 | 1.10 | 11.13 | 16.09 |  |  |  |  |  |  |  |  |
|  | Total | 49 | 12.84 | 3.87 | 0.55 | 11.73 | 13.96 |  |  |  |  |  |  |  |  |
| LEFT Ankle Dor/Pla (degree) | NotFall-LFOF | 12 | 4.88 | 1.58 | 0.46 | 3.88 | 5.89 | 3.84 | 0.016  (0.15) | 0.018  (0.54) | 0.932  (0.17) | 0.239  (0.37) | 0.084  (0.47) | 0.799  (0.16) | 0.543  (0.27) |
|  | NotFall-HFOF | 15 | 7.28 | 2.16 | 0.56 | 6.09 | 8.47 |  |  |  |  |  |  |  |  |
|  | Fall-LFOF | 12 | 5.38 | 1.42 | 0.41 | 4.48 | 6.27 |  |  |  |  |  |  |  |  |
|  | Fall-HFOF | 10 | 6.53 | 2.75 | 0.87 | 4.57 | 8.49 |  |  |  |  |  |  |  |  |
|  | Total | 49 | 6.07 | 2.19 | 0.31 | 5.45 | 6.70 |  |  |  |  |  |  |  |  |
| LEFT Pelvic Up/Dn (degree) | NotFall-LFOF | 12 | 2.29 | .53 | 0.15 | 1.96 | 2.63 | 1.66 | 0.188  (0.04) | - | - | - | - | - | - |
|  | NotFall-HFOF | 15 | 3.17 | 1.12 | 0.29 | 2.55 | 3.79 |  |  |  |  |  |  |  |  |
|  | Fall-LFOF | 12 | 2.66 | 1.32 | 0.38 | 1.82 | 3.50 |  |  |  |  |  |  |  |  |
|  | Fall-HFOF | 10 | 3.50 | 2.33 | 0.74 | 1.83 | 5.17 |  |  |  |  |  |  |  |  |
|  | Total | 49 | 2.90 | 1.43 | 0.20 | 2.49 | 3.31 |  |  |  |  |  |  |  |  |
| LEFT Hip Add/Abd (degree) | NotFall-LFOF | 12 | 5.73 | 2.88 | 0.83 | 3.90 | 7.56 | 0.72 | 0.543  (-0.02) | - | - | - | - | - | - |
|  | NotFall-HFOF | 15 | 5.63 | 2.67 | 0.69 | 4.15 | 7.10 |  |  |  |  |  |  |  |  |
|  | Fall-LFOF | 12 | 4.43 | 2.03 | 0.59 | 3.14 | 5.72 |  |  |  |  |  |  |  |  |
|  | Fall-HFOF | 10 | 5.45 | 1.97 | 0.62 | 4.04 | 6.86 |  |  |  |  |  |  |  |  |
|  | Total | 49 | 5.32 | 2.43 | 0.35 | 4.63 | 6.02 |  |  |  |  |  |  |  |  |
| LEFT Pelvic Int/Ext (degree) | NotFall-LFOF | 12 | 5.41 | 3.11 | 0.90 | 3.43 | 7.38 | 0.32 | 0.813  (-0.04) | - | - | - | - | - | - |
|  | NotFall-HFOF | 15 | 4.86 | 1.30 | 0.34 | 4.14 | 5.58 |  |  |  |  |  |  |  |  |
|  | Fall-LFOF | 12 | 4.55 | 1.98 | 0.57 | 3.29 | 5.81 |  |  |  |  |  |  |  |  |
|  | Fall-HFOF | 10 | 5.09 | 2.40 | 0.76 | 3.37 | 6.81 |  |  |  |  |  |  |  |  |
|  | Total | 49 | 4.97 | 2.19 | 0.31 | 4.34 | 5.59 |  |  |  |  |  |  |  |  |
| LEFT Hip Int/Ext (degree) | NotFall-LFOF | 12 | 5.72 | 5.18 | 1.49 | 2.43 | 9.01 | 17.86 | 0.000  (0.51) | 0.939  (0.09) | 0.062  (0.36) | 0.000  (0.73) | 0.157  (0.60) | 0.000  (0.99) | 0.001  (0.84) |
|  | NotFall-HFOF | 15 | 6.35 | 0.67 | 0.17 | 5.98 | 6.72 |  |  |  |  |  |  |  |  |
|  | Fall-LFOF | 12 | 8.68 | 2.27 | 0.66 | 7.24 | 10.13 |  |  |  |  |  |  |  |  |
|  | Fall-HFOF | 10 | 13.66 | 0.13 | 0.04 | 13.57 | 13.75 |  |  |  |  |  |  |  |  |
|  | Total | 49 | 8.26 | 4.04 | 0.58 | 7.10 | 9.42 |  |  |  |  |  |  |  |  |
| LEFT Foot Int/Ext (degree) | NotFall-LFOF | 12 | 6.33 | 2.43 | 0.70 | 4.79 | 7.88 | 1.67 | 0.187  (0.04) | - | - | - | - | - | - |
|  | NotFall-HFOF | 15 | 6.75 | 3.43 | 0.88 | 4.85 | 8.64 |  |  |  |  |  |  |  |  |
|  | Fall-LFOF | 12 | 7.26 | 3.09 | 0.89 | 5.29 | 9.22 |  |  |  |  |  |  |  |  |
|  | Fall-HFOF | 10 | 4.60 | 2.37 | 0.75 | 2.90 | 6.30 |  |  |  |  |  |  |  |  |
|  | Total | 49 | 6.33 | 2.99 | 0.43 | 5.48 | 7.19 |  |  |  |  |  |  |  |  |
| RIGHT Pelvis Ant/Pst (degree) | NotFall-LFOF | 12 | 3.83 | 3.36 | 0.97 | 1.69 | 5.96 | 1.41 | 0.254  (0.02) | - | - | - | - | - | - |
|  | NotFall-HFOF | 15 | 6.89 | 5.40 | 1.40 | 3.89 | 9.88 |  |  |  |  |  |  |  |  |
|  | Fall-LFOF | 12 | 4.44 | 4.09 | 1.18 | 1.84 | 7.04 |  |  |  |  |  |  |  |  |
|  | Fall-HFOF | 10 | 4.46 | 3.33 | 1.05 | 2.08 | 6.84 |  |  |  |  |  |  |  |  |
|  | Total | 49 | 5.04 | 4.31 | 0.62 | 3.80 | 6.28 |  |  |  |  |  |  |  |  |
| RIGHT Hip Flx/Ext (degree) | NotFall-LFOF | 12 | 8.52 | 4.69 | 1.35 | 5.54 | 11.49 | 0.95 | 0.423  (0.00) | - | - | - | - | - | - |
|  | NotFall-HFOF | 15 | 11.32 | 5.96 | 1.54 | 8.02 | 14.62 |  |  |  |  |  |  |  |  |
|  | Fall-LFOF | 12 | 8.52 | 5.23 | 1.51 | 5.19 | 11.84 |  |  |  |  |  |  |  |  |
|  | Fall-HFOF | 10 | 9.36 | 3.47 | 1.10 | 6.88 | 11.84 |  |  |  |  |  |  |  |  |
|  | Total | 49 | 9.55 | 5.05 | 0.72 | 8.10 | 11.00 |  |  |  |  |  |  |  |  |
| RIGHT Knee Flx/Ext (degree) | NotFall-LFOF | 12 | 9.53 | 3.70 | 1.07 | 7.18 | 11.88 | 2.00 | 0.128  (0.06) | - | - | - | - | - | - |
|  | NotFall-HFOF | 15 | 13.28 | 4.59 | 1.19 | 10.74 | 15.82 |  |  |  |  |  |  |  |  |
|  | Fall-LFOF | 12 | 11.42 | 3.99 | 1.15 | 8.88 | 13.95 |  |  |  |  |  |  |  |  |
|  | Fall-HFOF | 10 | 12.92 | 4.66 | 1.47 | 9.59 | 16.25 |  |  |  |  |  |  |  |  |
|  | Total | 49 | 11.83 | 4.39 | 0.63 | 10.57 | 13.09 |  |  |  |  |  |  |  |  |
| RIGHT Ankle Dor/Pla (degree) | NotFall-LFOF | 12 | 5.51 | 1.45 | 0.42 | 4.59 | 6.43 | 2.45 | 0.076  (0.08) | - | - | - | - | - | - |
|  | NotFall-HFOF | 15 | 6.61 | 2.47 | 0.64 | 5.24 | 7.98 |  |  |  |  |  |  |  |  |
|  | Fall-LFOF | 12 | 5.28 | 1.61 | 0.46 | 4.25 | 6.30 |  |  |  |  |  |  |  |  |
|  | Fall-HFOF | 10 | 7.11 | 1.65 | 0.52 | 5.93 | 8.29 |  |  |  |  |  |  |  |  |
|  | Total | 49 | 6.11 | 1.98 | 0.28 | 5.55 | 6.68 |  |  |  |  |  |  |  |  |
| RIGHT Pelvic Up/Dn (degree) | NotFall-LFOF | 12 | 2.29 | 0.53 | 0.15 | 1.96 | 2.63 | 1.66 | 0.188  (0.04) | - | - | - | - | - | - |
|  | NotFall-HFOF | 15 | 3.17 | 1.12 | 0.29 | 2.55 | 3.79 |  |  |  |  |  |  |  |  |
|  | Fall-LFOF | 12 | 2.66 | 1.32 | 0.38 | 1.82 | 3.50 |  |  |  |  |  |  |  |  |
|  | Fall-HFOF | 10 | 3.50 | 2.33 | 0.74 | 1.83 | 5.17 |  |  |  |  |  |  |  |  |
|  | Total | 49 | 2.90 | 1.43 | 0.20 | 2.49 | 3.31 |  |  |  |  |  |  |  |  |
| RIGHT Hip Add/Abd (degree) | NotFall-LFOF | 12 | 5.15 | 2.17 | 0.63 | 3.77 | 6.53 | 0.97 | 0.416  (0.00) | - | - | - | - | - | - |
|  | NotFall-HFOF | 15 | 6.62 | 2.73 | 0.70 | 5.11 | 8.13 |  |  |  |  |  |  |  |  |
|  | Fall-LFOF | 12 | 5.39 | 2.45 | 0.71 | 3.84 | 6.95 |  |  |  |  |  |  |  |  |
|  | Fall-HFOF | 10 | 5.50 | 2.44 | 0.77 | 3.75 | 7.25 |  |  |  |  |  |  |  |  |
|  | Total | 49 | 5.73 | 2.47 | 0.35 | 5.02 | 6.44 |  |  |  |  |  |  |  |  |
| RIGHT Pelvic Int/Ext (degree) | NotFall-LFOF | 12 | 5.41 | 3.11 | 0.90 | 3.43 | 7.38 | 0.32 | 0.813  (-0.04) | - | - | - | - | - | - |
|  | NotFall-HFOF | 15 | 4.86 | 1.30 | 0.34 | 4.14 | 5.58 |  |  |  |  |  |  |  |  |
|  | Fall-LFOF | 12 | 4.55 | 1.98 | 0.57 | 3.29 | 5.81 |  |  |  |  |  |  |  |  |
|  | Fall-HFOF | 10 | 5.09 | 2.40 | 0.76 | 3.37 | 6.81 |  |  |  |  |  |  |  |  |
|  | Total | 49 | 4.97 | 2.19 | 0.31 | 4.34 | 5.59 |  |  |  |  |  |  |  |  |
| RIGHT Hip Int/Ext (degree) | NotFall-LFOF | 12 | 6.57 | 4.67 | 1.35 | 3.60 | 9.54 | 11.06 | 0.000  (0.38) | 0.647  (0.26) | 0.421  (0.00) | 0.000  (0.10) | 0.969  (0.25) | 0.000  (0.19) | 0.001  (0.10) |
|  | NotFall-HFOF | 15 | 7.97 | 3.35 | 0.86 | 6.11 | 9.82 |  |  |  |  |  |  |  |  |
|  | Fall-LFOF | 12 | 8.51 | 1.64 | 0.47 | 7.47 | 9.55 |  |  |  |  |  |  |  |  |
|  | Fall-HFOF | 10 | 13.75 | 0.32 | 0.10 | 13.52 | 13.98 |  |  |  |  |  |  |  |  |
|  | Total | 49 | 8.94 | 3.93 | 0.56 | 7.81 | 10.07 |  |  |  |  |  |  |  |  |
| RIGHT Foot Int/Ext (degree) | NotFall-LFOF | 12 | 8.24 | 4.27 | 1.23 | 5.53 | 10.95 | 1.12 | 0.353  (0.01) | - | - | - | - | - | - |
|  | NotFall-HFOF | 15 | 6.02 | 2.57 | 0.66 | 4.60 | 7.44 |  |  |  |  |  |  |  |  |
|  | Fall-LFOF | 12 | 6.18 | 3.50 | 1.01 | 3.96 | 8.41 |  |  |  |  |  |  |  |  |
|  | Fall-HFOF | 10 | 6.71 | 3.17 | 1.00 | 4.44 | 8.98 |  |  |  |  |  |  |  |  |
|  | Total | 49 | 6.74 | 3.41 | 0.49 | 5.77 | 7.72 |  |  |  |  |  |  |  |  |
| ***Data Exposed*** |  |  |  |  |  |  |  |  |  |  |  |  |  |  |  |
| GPS (Left) (degree) | NotFall-LFOF | 12 | 8.88 | 1.51 | 0.44 | 7.92 | 9.83 | 3.17 | 0.033  (0.12) | 0.121  (0.37) | 0.944  (0.14) | 0.985  (0.11) | 0.032  (0.44) | 0.294  (0.31) | 0.815  (0.25) |
|  | NotFall-HFOF | 15 | 10.49 | 2.48 | 0.64 | 9.11 | 11.86 |  |  |  |  |  |  |  |  |
|  | Fall-LFOF | 12 | 8.46 | 1.62 | 0.47 | 7.43 | 9.49 |  |  |  |  |  |  |  |  |
|  | Fall-HFOF | 10 | 9.15 | 1.13 | 0.36 | 8.34 | 9.96 |  |  |  |  |  |  |  |  |
|  | Total | 49 | 9.32 | 1.96 | 0.28 | 8.76 | 9.88 |  |  |  |  |  |  |  |  |
| GPS (Right) (degree) | NotFall-LFOF | 12 | 8.51 | 1.61 | 0.47 | 7.48 | 9.53 | 1.47 | 0.237  (0.03) | - | - | - | - | - | - |
|  | NotFall-HFOF | 15 | 9.95 | 2.49 | 0.64 | 8.57 | 11.33 |  |  |  |  |  |  |  |  |
|  | Fall-LFOF | 12 | 8.46 | 2.14 | 0.62 | 7.10 | 9.82 |  |  |  |  |  |  |  |  |
|  | Fall-HFOF | 10 | 9.18 | 2.05 | 0.65 | 7.71 | 10.65 |  |  |  |  |  |  |  |  |
|  | Total | 49 | 9.07 | 2.16 | 0.31 | 8.45 | 9.69 |  |  |  |  |  |  |  |  |
| GPS (Overall) (degree) | NotFall-LFOF | 12 | 9.33 | 1.29 | 0.37 | 8.51 | 10.16 | 2.67 | 0.044  (0.10) | 0.139  (0.37) | 0.984  (0.09) | 0.907  (0.20) | 0.042  (0.41) | 0.517  (0.24) | 0.744  (0.25 |
|  | NotFall-HFOF | 15 | 10.89 | 2.44 | 0.63 | 9.54 | 12.24 |  |  |  |  |  |  |  |  |
|  | Fall-LFOF | 12 | 9.07 | 1.65 | 0.48 | 8.02 | 10.11 |  |  |  |  |  |  |  |  |
|  | Fall-HFOF | 10 | 9.86 | 1.48 | 0.47 | 8.80 | 10.92 |  |  |  |  |  |  |  |  |
|  | Total | 49 | 9.85 | 1.93 | 0.28 | 9.30 | 10.41 |  |  |  |  |  |  |  |  |
| LEFT Pelvis Ant/Pst (degree) | NotFall-LFOF | 12 | 4.00 | 3.23 | 0.93 | 1.95 | 6.05 | 1.23 | 0.308  (0.01) | - | - | - | - | - | - |
|  | NotFall-HFOF | 15 | 6.97 | 5.60 | 1.44 | 3.87 | 10.07 |  |  |  |  |  |  |  |  |
|  | Fall-LFOF | 12 | 4.84 | 4.46 | 1.29 | 2.01 | 7.68 |  |  |  |  |  |  |  |  |
|  | Fall-HFOF | 10 | 4.54 | 2.93 | 0.93 | 2.44 | 6.64 |  |  |  |  |  |  |  |  |
|  | Total | 49 | 5.22 | 4.38 | 0.63 | 3.97 | 6.48 |  |  |  |  |  |  |  |  |
| LEFT Hip Flx/Ext (degree) | NotFall-LFOF | 12 | 10.28 | 4.06 | 1.17 | 7.70 | 12.86 | 2.24 | 0.097  (0.07) | - | - | - | - | - | - |
|  | NotFall-HFOF | 15 | 13.71 | 7.71 | 1.99 | 9.44 | 17.99 |  |  |  |  |  |  |  |  |
|  | Fall-LFOF | 12 | 8.48 | 3.63 | 1.05 | 6.18 | 10.79 |  |  |  |  |  |  |  |  |
|  | Fall-HFOF | 10 | 11.39 | 3.90 | 1.23 | 8.60 | 14.18 |  |  |  |  |  |  |  |  |
|  | Total | 49 | 11.12 | 5.57 | 0.80 | 9.52 | 12.72 |  |  |  |  |  |  |  |  |
| LEFT Knee Flx/Ext (degree) | NotFall-LFOF | 12 | 15.80 | 4.66 | 1.35 | 12.84 | 18.76 | 0.23 | 0.878  (-0.05) | - | - | - | - | - | - |
|  | NotFall-HFOF | 15 | 15.01 | 6.62 | 1.71 | 11.34 | 18.67 |  |  |  |  |  |  |  |  |
|  | Fall-LFOF | 12 | 14.15 | 4.83 | 1.39 | 11.08 | 17.22 |  |  |  |  |  |  |  |  |
|  | Fall-HFOF | 10 | 15.42 | 2.95 | 0.93 | 13.31 | 17.53 |  |  |  |  |  |  |  |  |
|  | Total | 49 | 15.08 | 5.01 | 0.72 | 13.64 | 16.51 |  |  |  |  |  |  |  |  |
| LEFT Ankle Dor/Pla (degree) | NotFall-LFOF | 12 | 6.64 | 1.31 | 0.38 | 5.81 | 7.47 | 0.76 | 0.521  (-0.01) | - | - | - | - | - | - |
|  | NotFall-HFOF | 15 | 7.73 | 2.30 | 0.59 | 6.46 | 9.00 |  |  |  |  |  |  |  |  |
|  | Fall-LFOF | 12 | 6.73 | 2.14 | 0.62 | 5.37 | 8.08 |  |  |  |  |  |  |  |  |
|  | Fall-HFOF | 10 | 7.52 | 3.08 | 0.97 | 5.32 | 9.72 |  |  |  |  |  |  |  |  |
|  | Total | 49 | 7.17 | 2.24 | 0.32 | 6.53 | 7.82 |  |  |  |  |  |  |  |  |
| LEFT Pelvic Up/Dn (degree) | NotFall-LFOF | 12 | 2.68 | 0.67 | 0.19 | 2.26 | 3.11 | 1.69 | 0.183  (0.04) | - | - | - | - | - | - |
|  | NotFall-HFOF | 15 | 3.56 | 1.27 | 0.33 | 2.86 | 4.26 |  |  |  |  |  |  |  |  |
|  | Fall-LFOF | 12 | 2.62 | 1.36 | 0.39 | 1.75 | 3.48 |  |  |  |  |  |  |  |  |
|  | Fall-HFOF | 10 | 3.35 | 1.80 | 0.57 | 2.06 | 4.64 |  |  |  |  |  |  |  |  |
|  | Total | 49 | 3.07 | 1.33 | 0.19 | 2.69 | 3.45 |  |  |  |  |  |  |  |  |
| LEFT Hip Add/Abd (degree) | NotFall-LFOF | 12 | 6.03 | 3.42 | 0.99 | 3.86 | 8.21 | 0.29 | 0.834  (-0.05) | - | - | - | - | - | - |
|  | NotFall-HFOF | 15 | 6.05 | 2.41 | 0.62 | 4.71 | 7.38 |  |  |  |  |  |  |  |  |
|  | Fall-LFOF | 12 | 5.31 | 2.02 | 0.58 | 4.03 | 6.59 |  |  |  |  |  |  |  |  |
|  | Fall-HFOF | 10 | 6.23 | 2.32 | 0.73 | 4.57 | 7.89 |  |  |  |  |  |  |  |  |
|  | Total | 49 | 5.90 | 2.54 | 0.36 | 5.17 | 6.63 |  |  |  |  |  |  |  |  |
| LEFT Pelvic Int/Ext (degree) | NotFall-LFOF | 12 | 5.69 | 2.19 | 0.63 | 4.30 | 7.08 | 0.60 | 0.621  (-0.03) | - | - | - | - | - | - |
|  | NotFall-HFOF | 15 | 5.56 | 1.19 | 0.31 | 4.90 | 6.22 |  |  |  |  |  |  |  |  |
|  | Fall-LFOF | 12 | 5.07 | 1.90 | 0.55 | 3.86 | 6.28 |  |  |  |  |  |  |  |  |
|  | Fall-HFOF | 10 | 4.86 | 1.62 | 0.51 | 3.70 | 6.02 |  |  |  |  |  |  |  |  |
|  | Total | 49 | 5.33 | 1.72 | 0.25 | 4.84 | 5.82 |  |  |  |  |  |  |  |  |
| LEFT Hip Int/Ext (degree) | NotFall-LFOF | 12 | 10.38 | 3.73 | 1.08 | 8.01 | 12.74 | 2.47 | 0.074  (0.08) | - | - | - | - | - | - |
|  | NotFall-HFOF | 15 | 14.61 | 5.20 | 1.34 | 11.74 | 17.49 |  |  |  |  |  |  |  |  |
|  | Fall-LFOF | 12 | 11.68 | 3.36 | 0.97 | 9.55 | 13.82 |  |  |  |  |  |  |  |  |
|  | Fall-HFOF | 10 | 12.59 | 3.79 | 1.20 | 9.88 | 15.30 |  |  |  |  |  |  |  |  |
|  | Total | 49 | 12.44 | 4.36 | 0.62 | 11.19 | 13.70 |  |  |  |  |  |  |  |  |
| LEFT Foot Int/Ext (degree) | NotFall-LFOF | 12 | 6.69 | 3.13 | 0.90 | 4.70 | 8.68 | 1.39 | 0.258  (0.02) | - | - | - | - | - | - |
|  | NotFall-HFOF | 15 | 7.17 | 3.47 | 0.90 | 5.24 | 9.09 |  |  |  |  |  |  |  |  |
|  | Fall-LFOF | 12 | 6.53 | 3.19 | 0.92 | 4.51 | 8.56 |  |  |  |  |  |  |  |  |
|  | Fall-HFOF | 10 | 4.70 | 1.88 | 0.59 | 3.36 | 6.04 |  |  |  |  |  |  |  |  |
|  | Total | 49 | 6.39 | 3.09 | 0.44 | 5.50 | 7.28 |  |  |  |  |  |  |  |  |
| RIGHT Pelvis Ant/Pst (degree) | NotFall-LFOF | 12 | 4.00 | 3.23 | 0.93 | 1.95 | 6.05 | 1.23 | 0.308  (0.01) | - | - | - | - | - | - |
|  | NotFall-HFOF | 15 | 6.97 | 5.60 | 1.44 | 3.87 | 10.07 |  |  |  |  |  |  |  |  |
|  | Fall-LFOF | 12 | 4.84 | 4.46 | 1.29 | 2.01 | 7.68 |  |  |  |  |  |  |  |  |
|  | Fall-HFOF | 10 | 4.54 | 2.93 | 0.93 | 2.44 | 6.64 |  |  |  |  |  |  |  |  |
|  | Total | 49 | 5.22 | 4.38 | 0.63 | 3.97 | 6.48 |  |  |  |  |  |  |  |  |
| RIGHT Hip Flx/Ext (degree) | NotFall-LFOF | 12 | 8.91 | 4.47 | 1.29 | 6.07 | 11.75 | 1.75 | 0.170  (0.04) | - | - | - | - | - | - |
|  | NotFall-HFOF | 15 | 12.93 | 6.06 | 1.57 | 9.58 | 16.29 |  |  |  |  |  |  |  |  |
|  | Fall-LFOF | 12 | 9.36 | 5.37 | 1.55 | 5.95 | 12.77 |  |  |  |  |  |  |  |  |
|  | Fall-HFOF | 10 | 10.16 | 3.52 | 1.11 | 7.64 | 12.68 |  |  |  |  |  |  |  |  |
|  | Total | 49 | 10.51 | 5.20 | 0.74 | 9.01 | 12.00 |  |  |  |  |  |  |  |  |
| RIGHT Knee Flx/Ext (degree) | NotFall-LFOF | 12 | 12.59 | 4.80 | 1.38 | 9.54 | 15.64 | 1.85 | 0.151  (0.05) | - | - | - | - | - | - |
|  | NotFall-HFOF | 15 | 16.38 | 5.06 | 1.31 | 13.58 | 19.18 |  |  |  |  |  |  |  |  |
|  | Fall-LFOF | 12 | 12.69 | 4.36 | 1.26 | 9.92 | 15.46 |  |  |  |  |  |  |  |  |
|  | Fall-HFOF | 10 | 15.13 | 5.73 | 1.81 | 11.03 | 19.23 |  |  |  |  |  |  |  |  |
|  | Total | 49 | 14.29 | 5.11 | 0.73 | 12.83 | 15.76 |  |  |  |  |  |  |  |  |
| RIGHT Ankle Dor/Pla (degree) | NotFall-LFOF | 12 | 7.01 | 1.70 | 0.49 | 5.93 | 8.09 | 0.79 | 0.506  (-0.01) | - | - | - | - | - | - |
|  | NotFall-HFOF | 15 | 7.90 | 3.04 | 0.79 | 6.21 | 9.59 |  |  |  |  |  |  |  |  |
|  | Fall-LFOF | 12 | 6.73 | 1.81 | 0.52 | 5.57 | 7.88 |  |  |  |  |  |  |  |  |
|  | Fall-HFOF | 10 | 7.99 | 2.95 | 0.93 | 5.88 | 10.10 |  |  |  |  |  |  |  |  |
|  | Total | 49 | 7.41 | 2.46 | 0.35 | 6.71 | 8.12 |  |  |  |  |  |  |  |  |
| RIGHT Pelvic Up/Dn (degree) | NotFall-LFOF | 12 | 2.68 | 0.67 | 0.19 | 2.26 | 3.11 | 1.69 | 0.183  (0.04) | - | - | - | - | - | - |
|  | NotFall-HFOF | 15 | 3.56 | 1.27 | 0.33 | 2.86 | 4.26 |  |  |  |  |  |  |  |  |
|  | Fall-LFOF | 12 | 2.62 | 1.36 | 0.39 | 1.75 | 3.48 |  |  |  |  |  |  |  |  |
|  | Fall-HFOF | 10 | 3.35 | 1.80 | 0.57 | 2.06 | 4.64 |  |  |  |  |  |  |  |  |
|  | Total | 49 | 3.07 | 1.33 | 0.19 | 2.69 | 3.45 |  |  |  |  |  |  |  |  |
| RIGHT Hip Add/Abd (degree) | NotFall-LFOF | 12 | 6.06 | 2.21 | 0.64 | 4.65 | 7.46 | 0.82 | 0.491  (-0.01) | - | - | - | - | - | - |
|  | NotFall-HFOF | 15 | 7.10 | 2.64 | 0.68 | 5.64 | 8.56 |  |  |  |  |  |  |  |  |
|  | Fall-LFOF | 12 | 5.88 | 2.64 | 0.76 | 4.19 | 7.56 |  |  |  |  |  |  |  |  |
|  | Fall-HFOF | 10 | 5.91 | 1.79 | 0.57 | 4.63 | 7.19 |  |  |  |  |  |  |  |  |
|  | Total | 49 | 6.30 | 2.38 | 0.34 | 5.62 | 6.98 |  |  |  |  |  |  |  |  |
| RIGHT Pelvic Int/Ext (degree) | NotFall-LFOF | 12 | 5.69 | 2.19 | 0.63 | 4.30 | 7.08 | 0.60 | 0.621  (-0.02) | - | - | - | - | - | - |
|  | NotFall-HFOF | 15 | 5.56 | 1.19 | 0.31 | 4.90 | 6.22 |  |  |  |  |  |  |  |  |
|  | Fall-LFOF | 12 | 5.07 | 1.90 | 0.55 | 3.86 | 6.28 |  |  |  |  |  |  |  |  |
|  | Fall-HFOF | 10 | 4.86 | 1.62 | 0.51 | 3.70 | 6.02 |  |  |  |  |  |  |  |  |
|  | Total | 49 | 5.33 | 1.72 | 0.25 | 4.84 | 5.82 |  |  |  |  |  |  |  |  |
| RIGHT Hip Int/Ext (degree) | NotFall-LFOF | 12 | 11.89 | 3.18 | 0.92 | 9.87 | 13.91 | 0.33 | 0.803  (-0.04) | - | - | - | - | - | - |
|  | NotFall-HFOF | 15 | 12.18 | 3.42 | 0.88 | 10.29 | 14.07 |  |  |  |  |  |  |  |  |
|  | Fall-LFOF | 12 | 12.14 | 3.34 | 0.96 | 10.02 | 14.26 |  |  |  |  |  |  |  |  |
|  | Fall-HFOF | 10 | 13.19 | 2.93 | 0.93 | 11.10 | 15.28 |  |  |  |  |  |  |  |  |
|  | Total | 49 | 12.31 | 3.18 | 0.45 | 11.39 | 13.22 |  |  |  |  |  |  |  |  |
| RIGHT Foot Int/Ext (degree) | NotFall-LFOF | 12 | 8.28 | 3.91 | 1.13 | 5.80 | 10.77 | 1.17 | 0.330  (0.01) | - | - | - | - | - | - |
|  | NotFall-HFOF | 15 | 5.85 | 1.70 | 0.44 | 4.91 | 6.80 |  |  |  |  |  |  |  |  |
|  | Fall-LFOF | 12 | 7.07 | 3.83 | 1.10 | 4.64 | 9.50 |  |  |  |  |  |  |  |  |
|  | Fall-HFOF | 10 | 7.02 | 3.88 | 1.23 | 4.24 | 9.80 |  |  |  |  |  |  |  |  |
|  | Total | 49 | 6.98 | 3.37 | 0.48 | 6.02 | 7.95 |  |  |  |  |  |  |  |  |

Note: A - NotFall-LFOF; B-NotFall-HFOF; C-Fall-LFOF; D - FallHFOF. Data Not Exposed - data obtained before exposure to the fictional disturbing factor; Data Exposed - Data obtained during exposure to the fictional disturbing factor. Comparative analysis performed by ANOVA one way, considering the F ratio, effect size (ω) and significance of α≤0.05. Post Tukey post hoc analysis, considering effect size (r) and significance of α≤0.05.

**Table 3 -** Description and comparison of the maximum muscle strength of the lower limb muscle groups between the NotFall-LFOF, NotFall-HFOF, Fall-LFOF and Fall-HFOF groups.

|  | | N | Mean | Std. Deviation | Std. Error | 95% Confidence Interval for Mean | | F | *p* valor (ω²) | *post hoc* | | | | | |
| --- | --- | --- | --- | --- | --- | --- | --- | --- | --- | --- | --- | --- | --- | --- | --- |
|  |  |  |  |  |  | Lower Bound | Upper Bound |  |  | *A/B*  *(r)* | *A/C*  *(r)* | *A/D*  *(r)* | *B/C*  *(r)* | *B/D*  *(r)* | *C/D*  *(r)* |
| Hip abductors | NotFall-LFOF | 12 | 11.38 | 2.82 | 0.81 | 9.59 | 13.17 | 1.931 | 0.138  (0.05) | - | - | - | - | - | - |
|  | NotFall-HFOF | 15 | 10.55 | 2.49 | 0.64 | 9.17 | 11.93 |  |  |  |  |  |  |  |  |
|  | Fall-LFOF | 12 | 11.72 | 2.47 | 0.71 | 10.16 | 13.29 |  |  |  |  |  |  |  |  |
|  | Fall-HFOF | 10 | 9.45 | 1.41 | 0.44 | 8.44 | 10.46 |  |  |  |  |  |  |  |  |
|  | Total | 49 | 10.82 | 2.47 | 0.35 | 10.11 | 11.52 |  |  |  |  |  |  |  |  |
| Hip adductors | NotFall-LFOF | 12 | 10.72 | 2.15 | 0.62 | 9.35 | 12.08 | 1.983 | 0.130  (0.05) | - | - | - | - | - | - |
|  | NotFall-HFOF | 15 | 10.09 | 2.07 | 0.53 | 8.94 | 11.23 |  |  |  |  |  |  |  |  |
|  | Fall-LFOF | 12 | 11.03 | 2.54 | 0.73 | 9.42 | 12.64 |  |  |  |  |  |  |  |  |
|  | Fall-HFOF | 10 | 8.91 | 1.89 | 0.60 | 7.56 | 10.26 |  |  |  |  |  |  |  |  |
|  | Total | 49 | 10.23 | 2.24 | 0.32 | 9.59 | 10.87 |  |  |  |  |  |  |  |  |
| Hip extensors | NotFall-LFOF | 12 | 16.77 | 3.82 | 1.10 | 14.35 | 19.20 | 2.636 | 0.061  (0.12) | - | - | - | - | - | - |
|  | NotFall-HFOF | 15 | 13.99 | 4.84 | 1.25 | 11.31 | 16.66 |  |  |  |  |  |  |  |  |
|  | Fall-LFOF | 12 | 19.04 | 6.83 | 1.97 | 14.70 | 23.38 |  |  |  |  |  |  |  |  |
|  | Fall-HFOF | 10 | 14.23 | 4.65 | 1.47 | 10.91 | 17.56 |  |  |  |  |  |  |  |  |
|  | Total | 49 | 15.96 | 5.41 | 0.77 | 14.40 | 17.51 |  |  |  |  |  |  |  |  |
| Knee flexors | NotFall-LFOF | 12 | 15.04 | 4.32 | 1.25 | 12.30 | 17.79 | 1.489 | 0.230  (0.06) | - | - | - | - | - | - |
|  | NotFall-HFOF | 15 | 13.72 | 3.26 | 0.84 | 11.92 | 15.53 |  |  |  |  |  |  |  |  |
|  | Fall-LFOF | 12 | 16.95 | 4.15 | 1.20 | 14.32 | 19.59 |  |  |  |  |  |  |  |  |
|  | Fall-HFOF | 10 | 14.54 | 4.54 | 1.43 | 11.30 | 17.79 |  |  |  |  |  |  |  |  |
|  | Total | 49 | 15.00 | 4.08 | 0.58 | 13.83 | 16.18 |  |  |  |  |  |  |  |  |
| Plantiflexores | NotFall-LFOF | 12 | 17.87 | 3.69 | 1.06 | 15.53 | 20.21 | 2.809 | 0.050  (0.13) | 0.154  (0.27) | 0.948  (0.20) | 0.088  (0.50) | 0.154  (0.33) | 0.401  (0.12) | 0.242  (0.36) |
|  | NotFall-HFOF | 15 | 14.31 | 4.08 | 1.05 | 12.05 | 16.57 |  |  |  |  |  |  |  |  |
|  | Fall-LFOF | 12 | 16.93 | 4.62 | 1.33 | 13.99 | 19.86 |  |  |  |  |  |  |  |  |
|  | Fall-HFOF | 10 | 13.44 | 4.79 | 1.52 | 10.01 | 16.87 |  |  |  |  |  |  |  |  |
|  | Total | 49 | 15.64 | 4.52 | 0.65 | 14.35 | 16.94 |  |  |  |  |  |  |  |  |
| Dorsiflexores | NotFall-LFOF | 12 | 12.78 | 5.47 | 1.58 | 9.30 | 16.26 | 0.475 | 0.701  (-0.05) | - | - | - | - | - | - |
|  | NotFall-HFOF | 15 | 13.97 | 2.85 | 0.74 | 12.39 | 15.55 |  |  |  |  |  |  |  |  |
|  | Fall-LFOF | 12 | 15.10 | 5.69 | 1.64 | 11.48 | 18.72 |  |  |  |  |  |  |  |  |
|  | Fall-HFOF | 10 | 14.18 | 5.13 | 1.62 | 10.52 | 17.85 |  |  |  |  |  |  |  |  |
|  | Total | 49 | 14.00 | 4.72 | 0.67 | 12.64 | 15.35 |  |  |  |  |  |  |  |  |
| Hip flexors | NotFall-LFOF | 12 | 14.07 | 3.05 | 0.88 | 12.14 | 16.00 | 2.490 | 0.072  (0.10) | - | - | - | - | - | - |
|  | NotFall-HFOF | 15 | 13.70 | 4.22 | 1.09 | 11.36 | 16.03 |  |  |  |  |  |  |  |  |
|  | Fall-LFOF | 12 | 17.31 | 4.66 | 1.35 | 14.34 | 20.27 |  |  |  |  |  |  |  |  |
|  | Fall-HFOF | 10 | 13.64 | 3.11 | 0.98 | 11.42 | 15.87 |  |  |  |  |  |  |  |  |
|  | Total | 49 | 14.66 | 4.06 | 0.58 | 13.50 | 15.83 |  |  |  |  |  |  |  |  |
| Knee extensors | NotFall-LFOF | 12 | 21.00 | 4.96 | 1.43 | 17.85 | 24.15 | 2.765 | 0.053  (0.10) | - | - | - | - | - | - |
|  | NotFall-HFOF | 15 | 18.95 | 5.17 | 1.34 | 16.08 | 21.81 |  |  |  |  |  |  |  |  |
|  | Fall-LFOF | 12 | 25.36 | 8.30 | 2.40 | 20.09 | 30.63 |  |  |  |  |  |  |  |  |
|  | Fall-HFOF | 10 | 20.39 | 4.37 | 1.38 | 17.27 | 23.52 |  |  |  |  |  |  |  |  |
|  | Total | 49 | 21.32 | 6.23 | 0.89 | 19.53 | 23.11 |  |  |  |  |  |  |  |  |
| Medial hip rotators | NotFall-LFOF | 12 | 14.08 | 3.48 | 1.00 | 11.87 | 16.29 | 1.705 | 0.180  (0.10) | - | - | - | - | - | - |
|  | NotFall-HFOF | 15 | 13.18 | 4.06 | 1.05 | 10.93 | 15.43 |  |  |  |  |  |  |  |  |
|  | Fall-LFOF | 12 | 13.48 | 3.29 | 0.95 | 11.39 | 15.57 |  |  |  |  |  |  |  |  |
|  | Fall-HFOF | 10 | 10.74 | 3.61 | 1.14 | 8.16 | 13.32 |  |  |  |  |  |  |  |  |
|  | Total | 49 | 12.97 | 3.73 | 0.53 | 11.90 | 14.05 |  |  |  |  |  |  |  |  |
| Lateral hip rotators | NotFall-LFOF | 12 | 12.05 | 2.37 | 0.68 | 10.54 | 13.55 | 1.315 | 0.281  (0.11) | - | - | - | - | - | - |
|  | NotFall-HFOF | 15 | 10.52 | 3.03 | 0.78 | 8.84 | 12.20 |  |  |  |  |  |  |  |  |
|  | Fall-LFOF | 12 | 11.79 | 2.49 | 0.72 | 10.21 | 13.38 |  |  |  |  |  |  |  |  |
|  | Fall-HFOF | 10 | 10.45 | 1.87 | 0.59 | 9.12 | 11.79 |  |  |  |  |  |  |  |  |
|  | Total | 49 | 11.19 | 2.56 | 0.37 | 10.45 | 11.93 |  |  |  |  |  |  |  |  |

Note: A - NotFall-LFOF; B-NotFall-HFOF; C-Fall-LFOF; D-Fall-HFOF. Normalized muscle strength (kg force / kg body weight) x 100 (Piva, et al., 2005). Comparative analysis performed by ANOVA one way, considering the F ratio, effect size (ω) and significance of α≤0.05. Post Tukey post hoc analysis, considering effect size (r) and significance of α≤0.05.
